# Spatial distribution of heterogeneity as a modulator of collective dynamics in pancreatic beta-cell networks and beyond

**DOI:** 10.3389/fnetp.2023.1170930

**Published:** 2023-03-24

**Authors:** Daniel Galvis, David J. Hodson, Kyle C. A. Wedgwood

**Affiliations:** ^1^ Centre for Systems Modelling and Quantitative Biomedicine (SMQB), University of Birmingham, Birmingham, United Kingdom; ^2^ Institute of Metabolism and Systems Research (IMSR), University of Birmingham, Birmingham, United Kingdom; ^3^ Centre of Membrane Proteins and Receptors (COMPARE), University of Birmingham, Birmingham, United Kingdom; ^4^ Oxford Centre for Diabetes, Endocrinology, and Metabolism (OCDEM), Radcliffe Department of Medicine, Churchill Hospital, University of Oxford, Oxford, United Kingdom; ^5^ NIHR Oxford Biomedical Research Centre, Radcliffe Department of Medicine, Churchill Hospital, University of Oxford, Oxford, United Kingdom; ^6^ Living Systems Institute, University of Exeter, Exeter, United Kingdom; ^7^ EPSRC Hub for Quantitative Modelling in Healthcare, University of Exeter, Exeter, United Kingdom; ^8^ College of Engineering, Mathematics and Physical Sciences, University of Exeter, Exeter, United Kingdom

**Keywords:** excitable systems, collective dynamics, beta cell, sortedness, heterogeneous networks, phase transitions

## Abstract

We study the impact of spatial distribution of heterogeneity on collective dynamics in gap-junction coupled beta-cell networks comprised on cells from two populations that differ in their intrinsic excitability. Initially, these populations are uniformly and randomly distributed throughout the networks. We develop and apply an iterative algorithm for perturbing the arrangement of the network such that cells from the same population are increasingly likely to be adjacent to one another. We find that the global input strength, or *network drive*, necessary to transition the network from a state of quiescence to a state of synchronised and oscillatory activity decreases as *network sortedness* increases. Moreover, for weak coupling, we find that regimes of partial synchronisation and wave propagation arise, which depend both on network drive and network sortedness. We then demonstrate the utility of this algorithm for studying the distribution of heterogeneity in general networks, for which we use Watts–Strogatz networks as a case study. This work highlights the importance of heterogeneity in node dynamics in establishing collective rhythms in complex, excitable networks and has implications for a wide range of real-world systems that exhibit such heterogeneity.

## 1 Introduction

Many non-linear systems exhibit *excitable* behaviour, whereby they exhibit large-amplitude oscillations in response to small-amplitude, transient perturbations. One prominent example in biology is electrical excitability in cells, which underlies the function of neurons (Izhikevich, 2000; De Maesschalck and Wechselberger, 2015; Wedgwood et al., 2021), cardiac cells (Majumder et al., 2018; Barrio et al., 2020), pituitary cells (Sanchez-Cardenas et al., 2010; Hodson et al., 2012) and pancreatic beta cells (Bertram et al., 2007; McKenna et al., 2016). Moreover, the study of these systems is broadly applicable, and such excitable dynamics are also observed in semiconductor lasers (Terrien et al., 2020; Terrien et al., 2021), social media networks (Mathiesen et al., 2013), epidemiology (Vannucchi and Boccaletti, 2004), and wildfires (Punckt et al., 2015). When excitable units are combined into networks, they can generate complex rhythms (Bittihn et al., 2017; Fretter et al., 2017; Hörning et al., 2017). Interestingly, such networks may also generate dynamics that occur over low-dimensional manifolds of the full system (Ashwin and Swift, 1992; Watanabe and Strogatz, 1993; Ott and Antonsen, 2009; Bick et al., 2020). For example, neurons in the pre-Bötzinger complex fire synchronously to induce the inspiratory and expiratory phases during breathing (Wittmeier et al., 2008; Gaiteri and Rubin, 2011).

Heterogeneity is ubiquitous in natural systems. Whilst often portrayed as a undesirable attribute, it can play an important role in governing network dynamics (Manchanda et al., 2017; Delgado et al., 2018; Lambert and Vanni, 2018). For example, neurons may coarsely be stratified into excitatory and inhibitory groups, with the former promoting firing behaviour in other neurons and the latter suppressing it. When coupled, these neuronal subtypes give rise to a variety of behaviours, including synchronisation, and enable the network to respond differentially to incoming inputs (Börgers and Kopell, 2003; Börgers et al., 2005; Kopell et al., 2010). The classification of neuronal subtypes is becoming ever finer (Gouwens et al., 2019; Lipovsek et al., 2021) and it remains an open question as to how this heterogeneity governs overall brain dynamics. Even when networks comprise only a single unit type, heterogeneity may still impact the global dynamics. For example, if the natural frequencies of nodes in a coupled oscillator network are too far apart, the network will be unable to synchronise and will instead display more complex rhythms (Ottino-Löffler and Strogatz, 2016). In pancreatic islets, heterogeneity has increasingly been acknowledged as crucial for healthy glucose metabolism (Nasteska et al., 2021), and classification of beta-cell populations in particular is an area of active investigation. Heterogeneity in cell-intrinsic properties (i.e., excitability, metabolic activity, and genetic profiles), network properties, and functional properties have all been demonstrated (Johnston et al., 2016; Westacott et al., 2017; Nasteska et al., 2021; Kravets et al., 2022; Šterk et al., 2023).

Here, we explore transitions between quiescent states and collective oscillations in coupled networks of heterogeneous, excitable nodes. For the node dynamics, we treat two types of system, the first being the FitzHugh–Nagumo (FHN) model, which is a prototypical model of electrical excitability in biological cells (Fitzhugh, 1961; Nagumo, 1962), is often used to investigate collective network dynamics, and has been used as a model for beta cells (Scialla et al., 2021; Pedersen et al., 2022). For the second, we consider a conductance-based model of pancreatic beta cells designed to more closely reproduce the signalling dynamics in these cells (Sherman et al., 1988). These cells remain at rest until they receive a significantly large electrical impulse or, in the case of beta cells, the extracellular concentration of glucose surpasses a threshold value (Ashcroft Frances M and Rorsman, 1989; Braun et al., 2008).

For the past 40 years, based on empirical evidence from rodent islets, it has generally been assumed that beta cells form a *syncticium*, in which activity of an entire islet of Langerhans can be described by considering the dynamics of a single cell (Dolenšek et al., 2013; Podobnik et al., 2020; Satin et al., 2020). A number of studies have challenged this perspective in recent years, in particular highlighting that some ‘leader cells’ disproportionately influence the activity of the entire network which is made up primarily of ‘follower cells’ (Johnston et al., 2016; Westacott et al., 2017; Salem et al., 2019; Benninger and Kravets, 2021). In particular, one hypothesis suggests that beta cells can be grouped into two overarching classes based on their degree of excitability. This then suggests that islets are composed of a small number (∼10%) of highly excitable cells, with the remainder being less excitable (Benninger and Hodson, 2018).

To this end, we diffusively couple cells having two different degrees of excitability according to two different network architecture classes. The first of these comes from the Watts–Strogatz model, which allows for the generation of connected graphs, with consistent mean degree, and a parameter for adjusting the balance between local and long-range connections. This form of coupling allows one to explore architectures ranging from regular to small world graphs and, as such, is well-suited to investigations of the general types of graphs that occur in the natural world (Watts and Strogatz, 1998). While these graphs are not directly relevant to the electrical coupling architecture of pancreatic beta-cell networks, there is potential that the dynamics of beta-cell networks share common features with small-world networks. For example, small-worldness has be observed in the functional connectivity of pancreatic beta-cell Ca^2+^ signals (Stožer et al., 2013), and long-range connections have been proposed in mathematical modelling studies (Barua and Goel, 2016). The second model architecture was designed to mimic the spatial configuration of electrical coupling (i.e., connexin36 gap junctions) within beta-cell networks. Cells are locally coupled *via* gap junctions (Benninger et al., 2011; Rorsman and Braun, 2013) and spatially arranged in a three-dimensonal lattice embedded within a sphere.

With these network structures in mind, we explore how the spatial organisation of the two subpopulations affects the propensity of the whole network to oscillate in a synchronous fashion. The work of Westacott et al. (2017) recently demonstrated that *β*-cells with similar electrical excitability are spatially correlated within pancreatic islets. Motivated by this finding, we develop a metric for evaluating the degree of this spatial non-uniformity in *β*-cell networks and an algorithm for generating non-uniform networks with a specified value of this metric. We demonstrate that spatial correlation in heterogeneity, or *network sortedness*, plays a critical role in modulating collective dynamics in these networks. Moreover, we show that these metrics and algorithms can be applied to networks that do not have an explicit notion of space (e.g., Watts–Strogatz networks) and that network sortedness is similarly important in defining collective dynamics on these general network architectures. This work aims to demonstrate the importance of cellular organisation when studying heterogeneity in pancreatic *β*-cell networks (and in networks more generally) through a case study in heterogeneous excitability. Furthermore, it provides a set of tools for introducing correlated heterogeneities in arbitrary networks and studying their impact on dynamics. This spatial organisation is likely to be of particular relevance to the study of human islets, since these have been shown possess a highly non-trivial architecture, particularly when compared with rodent islets, on which most of our knowledge of beta cell function is based (Cabrera et al., 2006).

The remainder of the manuscript is arranged as follows: In Section 2, we describe the single cell dynamics and graph structures, introduce a metric that captures how sorted a network is with respect to its heterogeneity, and present an algorithm that can generate networks with arbitrary sortedness. In Section 3, we investigate how dynamic transitions to synchronous activity depend on the degree of sortedness in the network and end in Section 4 with concluding remarks.

## 2 Methods

### 2.1 Mathematical model

#### 2.1.1 Fitzhugh-Nagumo model (FHN)

The Fitzhugh-Nagumo model is a minimal model of an excitable unit. It exhibits relaxation oscillations when the external stimulus parameter, *I*, exceeds a critical value, where a Hopf bifurcation occurs. Here, we consider a network of *N* diffusively-coupled excitable nodes, which may, for example, be cells, each of which is described by the following model.

$$\frac{dV_{i}}{dt}=V_{i}-\frac{V_{i}^{3}}{3}-W_{i}+GI-I_{\mathrm{coup,i}},\;i=1,\ldots N,$$ (1)

$$\tau \frac{dW_{i}}{dt}=V_{i}+a-bW_{i}.$$ (2)

Here, *V* models the fast upstroke characteristic of excitability whilst *W* models the slow recovery (i.e., negative feedback). We replace the regular stimulus current term, *I*, with *GI*, where *I* is now the maximum stimulus current to a node, and thus defines the excitability of the node, whereas *G* ∈ [0, 1] is the degree of drive to the network. This allows us to consider a heterogeneous network model based on the excitability of nodes, but whilst including a network-wide drive parameter. A list of parameters is included in Supplementary Table S1.

#### 2.1.2 Sherman-Rinzel-Keizer model (SRK)

The electrical activity of pancreatic beta cells is proportional to the extracellular concentration of glucose. For sufficiently high extracellular glucose, the cells exhibit *bursting* dynamics, in which their voltage periodically switches between high frequency oscillations and quiescence. The high frequency oscillations in voltage are correlated with the secretion of insulin from these cells, so that these bursting dynamics are tightly coupled to the cells’ functional role. Here, we consider a network of *N* diffusively-coupled excitable cells, each of which is described by the three-variable model.

$$C_{m}\frac{dV_{i}}{dt}=-I_{K}\left(V_{i},n_{i}\right)-I_{Ca}\left(V_{i}\right)-I_{K-Ca}\left(V_{i},c_{i}\right)-I_{L}\left(V_{i}\right)-I_{\mathrm{coup,i}},\;i=1,\ldots N,$$ (3)

$$\frac{dn_{i}}{dt}=\frac{n_{\infty }\left(V_{i}\right)-n_{i}}{\tau_{n}\left(V_{i}\right)},$$ (4)

$$\frac{dc_{i}}{dt}=-f\left(\alpha I_{Ca}\left(V_{i}\right)+k_{Ca}c_{i}\right).$$ (5)

The variable *V* represents the membrane voltage of a *β*-cell, *n* is the activation variable for K^+^ ion channels, and *c* is the cytosolic Ca^2+^. This system was adapted from the Sherman–Rinzel–Keizer model, which describes the dynamics of electrical activity in pancreatic *β*-cells in the presence of glucose (Sherman et al., 1988). The intrinsic dynamics of the voltage, *V* given by Eq. 3 are driven by K^+^ (*I*_*K*_), Ca^2+^ (*I*_*Ca*_), and Ca^2+^-activated K^+^ (*I*_*K*−*Ca*_) ionic currents, with a rate governed by the whole cell capacitance given by *C*_*m*_. These currents are described *via*.

$$I_{K}\left(V,n\right)={\bar{g}}_{K}n\left(V-V_{k}\right),$$ (6)

$$I_{Ca}\left(V\right)={\bar{g}}_{Ca}m_{\infty }\left(V\right)h_{\infty }\left(V\right)\left(V-V_{Ca}\right),$$ (7)

$$I_{K-Ca}\left(V,c\right)={\bar{g}}_{K-Ca}\frac{c}{K_{d}+c}\left(V-V_{k}\right),$$ (8)

$$I_{L}\left(V\right)={\bar{g}}_{L}\left(1-G\right)\left(V-V_{K}\right).$$ (9)

In Eqs 6–9, ${\bar{g}}_{X}$ denotes the maximal conductance of the channel *X* where *X* ∈ {*K*, *Ca*, *K* − *Ca*, *L*} where *L* signifies a leak channel; *V*_*X*_ are the reversal potentials of the respective channels, *m* and *n* are the proportion of open activating gates for the Ca^2+^ and K^+^ channels, respectively; *h* is the proportion of open inactivating Ca^2+^ channels; *c* is the cytosolic concentration of Ca^2+^; and *G* is the extracellular concentration of glucose, which provides a global drive to promote activity and is taken to be homogeneous across the network. The activation of *I*_*K*−*Ca*_ is a function of free intracellular Ca^2+^ concentration and is defined by a Hill-type function with disassociation constant *K*_*d*_. The current *I*_*coup,i*_ captures the influence of the coupling between cells and will be discussed in Section 2.2.4.

The dynamics for *n*, *m*, and *h* follow exponential decay to their state values given by

$$x_{\infty }\left(V\right)=\frac{1}{1+\exp\left[\left(V_{x}-V\right)/S_{x}\right]},\;x\in \left\{h,m,n\right\},$$ (10)

at a rate given by the voltage-dependent time constant.

$$\tau_{n}\left(V\right)=\frac{\bar{\tau }}{\exp\left[\left(V-\bar{V}\right)/\kappa_{1}\right]+\exp\left[-\left(V-\bar{V}\right)/\kappa_{2}\right]}$$ (11)

for *n*. In Eq. 10, *V*_*x*_ represents the activation (inactivation) thresholds for *m* and *n* (*h*) and *S*_*x*_ represents the sensitivity of the channels around this point. Finally, Eq. 5 describes the evolution of the concentration of cytosolic Ca^2+^, which decays and is pumped out of the cell following a combined linear process with rate *k*_*Ca*_ and enters the cell *via* the Ca^2+^ ion channel at a rate given by the scale factor *α*. The parameter *f* specifies the fraction of free to bound Ca^2+^ in the cell, where bound Ca^2+^ plays no role in the relevant dynamics in our model.

**FIGURE 1:**
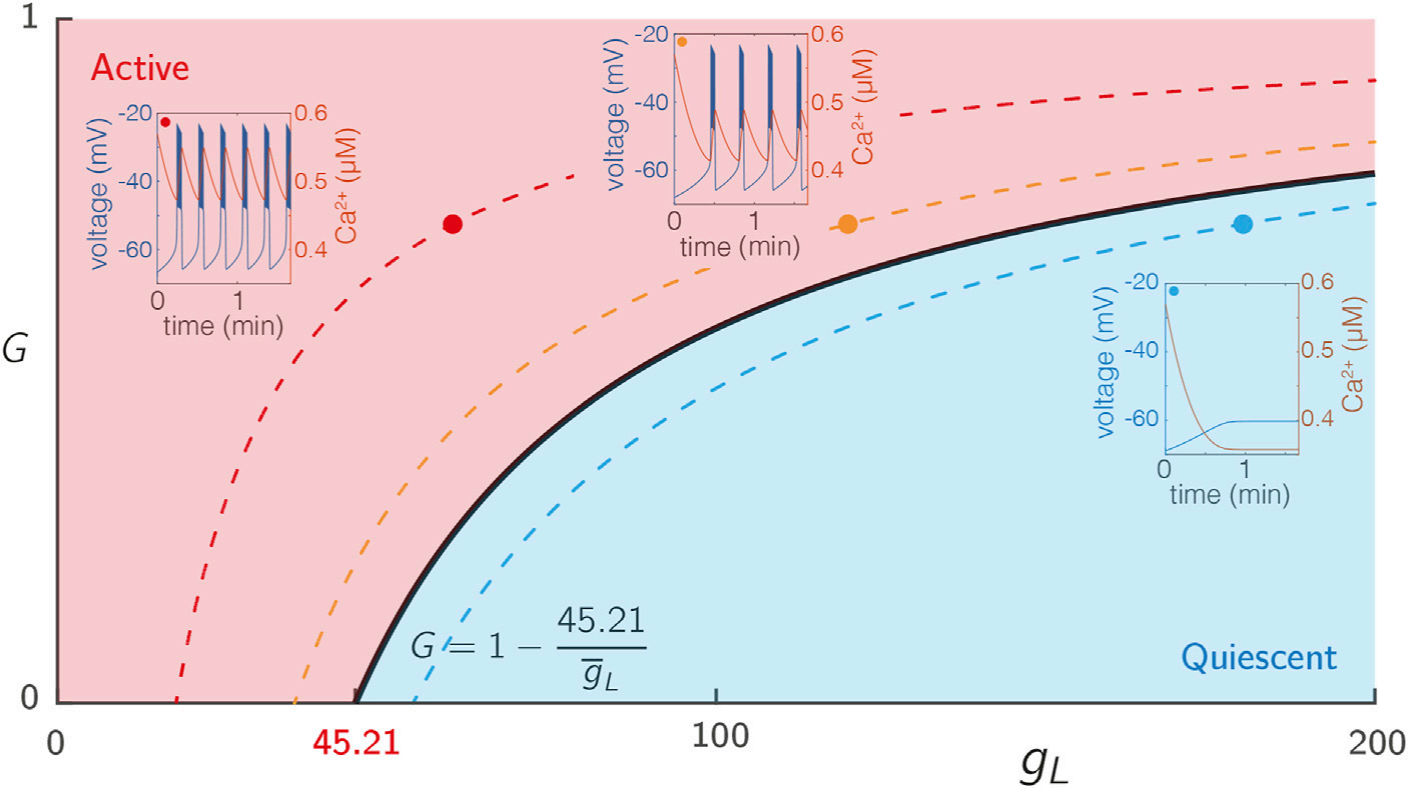
Excitability of single SRK cells. The voltage traces from 3 cells with varying levels of intrinsic excitability $\left({\bar{g}}_{L}\right)$ , but the same level of drive (*G* = 0.7). The red, blue, and black traces show decreasing levels of excitability with values of ${\bar{g}}_{L}=60$ , 120, and 180, respectively. More excitable cells have a shorter interburst interval. The solid black line represents a Hopf bifurcation as a function of both *G* and *g*_*L*_. At the lowest drive (*G* = 0), the Hopf bifurcation occurs for ${\bar{g}}_{L}=45.21$ pS. The dotted lines represent “level sets” of the $\left({\bar{g}}_{L},G\right)$ parameter space, along which the behaviour of the single cell is identical. Data for the bifurcation diagram was computed using XPP 8.0 (Ermentrout, 2002).

To expose the dependence of our system on glucose, we introduced a hyperpolarising leak current given by Eq. 9 that explicitly depends on the glucose concentration *G*. For an isolated cell (i.e., without coupling) with the parameters specified in Supplementary Table S2, the system describing each node exhibits steady state behaviour for low *G* and passes through a bifurcation as *G* ∈ [0, 1] is increased, as shown in Figure 1.

The bursting dynamics in our model are of the *fold-homoclinic* type under the classification specified in (Izhikevich, 2000). This classification is based on separation of the full system into a fast subsystem (Eqs 3, 4) and a slow subsystem (Eq. 5), treating the slow subsystem variables (in this case, *c*) as parameters in the fast subsystem. During each bursting cycle, the slow evolution of *c* pushes the fast subsystem through bifurcations that initiate and terminate oscillatory behaviour. In particular, when *c* decreases to a small enough value, the fast subsystem passes through a fold bifurcation in which a stable steady state and a saddle steady state collide and annihilate one another. Following this, the system exhibits stable periodic activity, during which *c* increases according to Eq. 5. When *c* increases to a sufficiently large value, the fast subsystem passes through a homoclinic bifurcation that destroys the periodic orbit and the system returns to the original stable steady state. Following this, *c* decreases until it once again reaches the fold point and the cycle repeats.

### 2.2 Model simulations

Simulations were conducted using Matlab 2019B. The dynamical systems were solved using ode15s, the relative tolerance set to 10^–5^, and explicit Jacobians were provided. The code was run on the University of Birmingham BlueBEAR HPC running RedHat 8.3 (x86_64) (see http://www.birmingham.ac.uk/bear for more details). Each set of simulations ran over 16 cores using a maximum of 128 GB RAM (32 GB was sufficient in most cases). All code used in the project is freely available for download from: github.com/dgalvis/network_spatial.

#### 2.2.1 Initial conditions

For the FHN model, the initial conditions $y_{i}\left(0\right)=\left(V_{i}\left(0\right),W_{i}\left(0\right)\right)$ for node *i* = 1, *…* , *N* were sampled independently from the distributions.

$$V_{i}\left(0\right)∼N\left(-1,{\left(1/6\right)}^{2}\right),\;W_{i}\left(0\right)=0.$$ (12)

For the SRK model, the initial conditions $y_{i}\left(0\right)=\left(V_{i}\left(0\right),n_{i}\left(0\right),c_{i}\left(0\right)\right)$ for node *i* = 1, *…* , *N* were sampled independently from the distributions

$$V_{i}\left(0\right)∼N\left(-68,{\left(68/6\right)}^{2}\right),\;n_{i}\left(0\right)=0,\;c_{i}\left(0\right)∼N\left(0.57,{\left(0.57/6\right)}^{2}\right).$$ (13)

The term $N\left(\mu ,\sigma^{2}\right)$ represents a normal distribution with mean *μ* and variance *σ* ^2^. Throughout, we use *Y* (0) to denote the set of initial conditions across the whole network model, i.e., $Y\left(0\right)=\left(y_{1}\left(0\right),\ldots ,y_{N}\left(0\right)\right)$ .

#### 2.2.2 Excitability and drive in the single-cell FHN model

The term *GI* is used to represent node excitability (*I*) and network drive (*G*). A Hopf bifurcation occurs at *GI* = 0.33. During the study, we partition the nodes into two sub-populations based on their excitability, with one population being highly excitable and the other being significantly less so.

#### 2.2.3 Excitability and drive in the single-cell SRK model

The ionic current *I*_*L*_ (Eq. 9) is a hyperpolarising current that can be used to adjust the excitability of each cell and to determine the activation level of the network model. In particular, the maximum conductance ${\bar{g}}_{L}$ determines the excitability of a cell. As this value increases, the cell becomes less excitable, that is, for a given value of *G*, cells with higher ${\bar{g}}_{L}$ are less likely to burst. This behaviour is summarised in Figure 1, which shows a two parameter bifurcation diagram showing the transition from quiescent to bursting behaviour under simultaneous variation of $\left({\bar{g}}_{L},G\right)$ , which occurs *via* a Hopf bifurcation of the full system (Eqs 3–5). For *G* = 0, this Hopf bifurcation occurs at ${\bar{g}}_{L}=45.21$ pS. For non-zero values of *G*, the bifurcation curve is defined *via* $\left(1-G\right){\bar{g}}_{L}=45.21$ pS, as can be seen by examining the form of the Eqs 3, 9. Note that when *G* = 1, system (Eqs 3–5) matches that of (Sherman et al., 1988). We use the observations about the link between ${\bar{g}}_{L}$ and excitability to partition the network into two sub-populations, one being highly excitable, the other being significantly less excitable.

#### 2.2.4 Graph structure and coupling

In this work, we consider two types of graph structure. The first is the Watts-Strogatz model of graph generation to create small-world graphs (WS graphs). We use *N* = 1000 nodes, a mean degree of *D* = 12, and unless stated, we use a rewiring probability of *β* = 0.2. The second type is motivated by pancreatic *β*-cell networks, which are arranged into roughly spherical clusters called islets of Langerhans (which also encompass other cell types that are disregarded in our model), which each contain ∼ 1,000 *β*-cells. To capture this, we arrange *N* = 1, 018 nodes on a hexagonal close packed (hcp) lattice embedded within a sphere (*β*C graph). Each node is connected to all of its nearest-neighbours so that the number of connections of nodes away from the boundary of the sphere is equal to the coordination number 12 whilst nodes on the boundary have fewer connections. This type of connectivity scheme is commonly employed in *β*-cell network modelling studies (Benninger et al., 2014; Westacott et al., 2017; Dwulet et al., 2019, 2021), as it qualitatively captures the network architecture. In reality, pancreatic islet architecture is more complex in terms of islet shape, cell shape, and the layout of cells in the tissue (Stožer et al., 2013; Šterk et al., 2023); all of which could be considered as additional sources of heterogeneity. Moreover, there may be heterogeneity in the number of connections that *β*-cells form amongst each other (Cabrera et al., 2006). We choose to employ this homogeneous lattice structure so that we could consider heterogeneity in cell-intrinsic excitability alone without introducing additional sources of heterogeneity due to the graph structure.

The dominant form of coupling between beta cells in the islets is through gap junctions, which allow small molecules, including charged ions to pass directly from a cell to its adjacent neighbours. Mathematically, this is represented through the inclusion of the diffusive term *I*_*coup,i*_ in Eq. 1 or Eq. 3 (we use the same coupling for both FHN and SRK models) given by

$$I_{\mathrm{coup,i}}={\bar{g}}_{\mathrm{coup}}\underset{j\in J_{i}}{\sum }\left(V_{i}-V_{j}\right),$$ (14)

where *J*_*i*_ is the set of all nodes to which node *i* is coupled.

Together, the two models for node dynamics (FHN and SRK models) and the two types of graph structure (WS and *β*C graphs) give rise to four combinations of network models, which we denote by WS-FHN, WS-SRK, *β*C-FHN, and *β*C-SRK. Note that throughout this work, we use the term graph to refer specifically to the set of links between nodes. We use the term network or network model to refer to the entirety of the model including choice of dynamics on the node (FHN or SRK), connectivity structure, and choice of parameters.

#### 2.2.5 Heterogeneity

For the FHN model, we consider networks consisting of two sub-populations of nodes distinguished by their excitability (i.e., by their *I* values). Population 1 is highly excitable (*I* = 2) and population 2 is less excitable (*I* = 1). Similarly for the SRK model, we consider networks consisting of two sub-populations of nodes distinguished by their excitability (i.e., by their ${\bar{g}}_{L}$ values). Population 1 is highly excitable ( ${\bar{g}}_{L}=60$ pS) and population 2 is less excitable ( ${\bar{g}}_{L}=100$ pS). We then consider the range over *G* for which population 1 nodes are intrinsically active (i.e., *G* such that population 1 is active when ${\bar{g}}_{\mathrm{coup}}=0$ ). We then consider the effects of degree of sortedness between the two subpopulations (see Section 2.3.1), graph structure (WS or *β*C), global network drive (*G*), and global coupling strength $\left({\bar{g}}_{\mathrm{coup}}\right)$ on the collective dynamics of the network.

### 2.3 Measuring sortedness

#### 2.3.1 Definition

To track the degree of sortedness in the network, we define a *node sortedness* measure that, for a given node, measures the proportion of neighbours that are of the same population type. For a general network with nodes attributed to K∈N populations, the node sortedness, *A*_*i*_, is defined as

$$A_{i}=\frac{1}{\left|J_{i}\right|}\underset{j\in J_{i}}{\sum }\chi_{ij},\;\chi_{ij}=\overset{K}{\underset{k=1}{\sum }}\mu_{i}^{\left(k\right)}\mu_{j}^{\left(k\right)},\;i=1,\ldots ,N,\;\mu_{i}^{\left(k\right)}=\left\{\begin{array}{cc} 1,\; & i\in P_{k}\\ 0,\; & \text{otherwise} \end{array}\right.,$$ (15)

where the *population sets* *P*_*k*_ contain the indices of the nodes within population *k* = 1, *…* , *K* and form a partition over the node indices {1, 2, *…* , *N*}, *J*_*i*_ is the set of indices of nodes that are coupled to node *i*, $\mu_{i}^{\left(k\right)}$ is an indicator function that takes value 1 if *i* belongs to population *k* and value 0 otherwise, and *χ*_*ij*_ is an indicator function that takes value 1 when node *i* and *j* belong to the same population and value 0 otherwise. For each population, the average node sortedness is defined *via*.

$${\bar{A}}_{k}=\frac{1}{\left|P_{k}\right|}\underset{n\in P_{k}}{\sum }A_{n},\;k=1,2,\ldots K.$$ (16)

Finally, the *network sortedness* is defined as

$$A=\frac{1}{K-1}\left(-1+\overset{K}{\underset{k=1}{\sum }}{\bar{A}}_{k}\right).$$ (17)

where A∈[−1/(K−1),1] and, for the present case with *K* = 2, A∈[−1,1] . For a network in which populations are assigned to nodes following a uniformly random distribution, A≈0 since ${\bar{A}}_{k}$ is approximately equal to *N*_*k*_/*N* where *N*_*k*_, *k* = 1, 2 is the number of nodes in population *k*. And therefore $\sum_{k}{\bar{A}}_{k}\approx 1$ . An illustration of the computation of the sortedness metrics (Eqs 15–17) is shown in Supplementary Figure S1A.

#### 2.3.2 Modified network sortedness for beta cell networks

For a *β*C lattice network, ${\bar{A}}_{k}$ (as defined in Eq. 16) is maximised when population 1 nodes are arranged in a single cluster with a minimal number of connections to population 2 nodes. This naturally occurs at the edges of the domain, since any cluster of population 1 nodes in the domain interior must be surrounded by population 2 nodes. We are interested in the dynamics that arise as the small population of highly excitable nodes forms clusters within the lattice, hence, we wish to remove this tendency for clusters to form at the domain boundary. To overcome this, we use a modified definition of the node sortedness (Eq. 15).

$${\tilde{A}}_{i}=\frac{1}{J}\underset{j\in J_{i}}{\sum }\chi_{ij}+\frac{\mu_{i}^{\left(2\right)}\left(J-|J_{i}|\right)}{J},\;i=1,\ldots N,$$ (18)

where *J* = 12 is the number of connections that interior lattice nodes possess. For nodes with |*J*_*i*_| < *J* (i.e., nodes on the domain boundary) the additional term in Eq. 18 compared to Eq. 15 incorporates a further *J* − |*J*_*i*_| connections to population 2 nodes for the purposes of calculating node sortedness values. This procedure is equivalent to assuming that the lattice defining our domain is embedded within a larger lattice of population 2 nodes.

### 2.4 Modifying network sortedness

#### 2.4.1 The sorting algorithm

Here, we describe our approach for generating networks with different network sortedness. The algorithm works by exchanging the population type of nodes from different populations randomly to increase (or decrease) A . The algorithm begins by randomly permuting the order of the *N* indices. The first *N*_1_ indices of the permuted sequence are attributed to *P*_1_, with the remaining *N*_2_ indices attributed to *P*_2_, yielding a distribution of population 1 nodes that is uniformly random in space.

On each iteration, *a*, of the algorithm, pairs of nodes (from different populations) are sampled without replacement from a joint probability density function (pdf) *P*(*X* = *i*, *Y* = *j*) = *f* (*i*, *j*), *i* ∈ *P*_1_, *j* ∈ *P*_2_, where *X* and *Y* are random integer variables indicating the node selected from population 1 and 2, respectively. The population types of these nodes are then exchanged, that is, if *i* ∈ *P*_1_ and *j* ∈ *P*_2_, then *i* is added to *P*_2_ and removed from *P*_1_ and *vice versa* for *j*. The network sortedness (Eq. 17) is then recomputed for the adjusted population sets. If the exchange leads to an increase (decrease) in A , the exchange is accepted and the algorithm proceeds to iteration *a* + 1. If the exchange does not lead to an increase (decrease) in A , the exchange is rejected and indices *i* and *j* are placed back in *P*_1_ and *P*_2_, respectively. In this case, a new pair of nodes is drawn from *f* and the process is repeated until either: a pair whose exchange leads to an increase (decrease) in A is found and the algorithm proceeds to the next iteration; or it is determined that no such pair exists, at which point the algorithm terminates. An example of one iteration of this algorithm is depicted in Supplementary Figure S1B. We refer to the algorithm in which swaps are accepted only if they lead to an increase (decrease) in A as the *forward* (*backward*) algorithm. We define $A_{a}$ to be the evaluation of A of the network after *a* iterations, where *a* ∈ {0 … *a*_*final*_}. Running the algorithm to convergence (*a*_*final*_) produces the sets $P_{k}={\left\{P_{k}^{a}\right\}}_{a=0}^{a_{\text{final}}}$ containing the population sets after each iteration.

**FIGURE 2:**
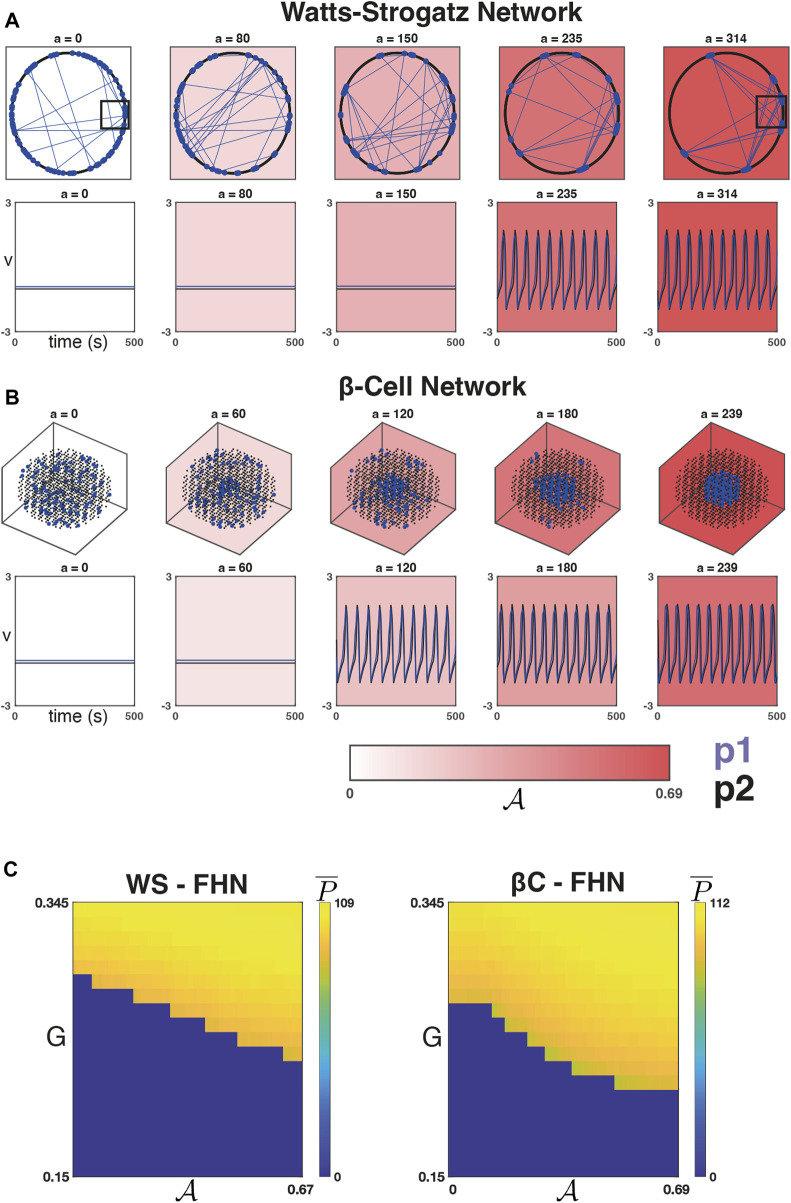
Dynamics on networks defined by the sorting algorithm. **(A)** *Top Row:* Five iterations of the sorting algorithm on a WS graph (with *β* = 0.2, *D* = 12). The majority of connections in this graph (≈80%) are between a node and its *D*/2 = 6 closest neighbours in each direction along the ring. The other ≈20% of connections are randomly selected for rewiring. In each panel, only the connections within population 1 (blue lines) are shown, and only the rewiring connections are visible. As A increases, the population 1 nodes (blue points) begin to form clusters with many connections between clusters. The black boxes are shown zoomed in on Supplementary Figure S1C. *Bottom Row:* The dynamics of *V* (WS-FHN) averaged across population 1 (blue) and 2 (black) are shown for a fixed value of *G* = 0.253, for strong coupling ${\bar{g}}_{\mathrm{coup}}=0.1$ , and on each of the five iterations of the sorting algorithm. A phase transition from quiescence to global, synchronised activity occurs with respect to A . **(B)** *Top Row:* Five iterations of the sorting algorithm on the *β*-cell graph. Initially, the population 1 cells (blue) are spread uniform randomly throughout the lattice. As A increases, they begin to form localised clusters culminating in a single cluster at convergence (*a* = 239). *Bottom Row:* The dynamics of *V* (*β*C-FHN) averaged across population 1 (blue) and 2 (black) are shown for a fixed value of *G* = 0.253, for strong coupling ${\bar{g}}_{\mathrm{coup}}=0.1$ , and on each of the five iterations of the sorting algorithm. A phase transition from quiescence to global, synchronised activity again occurs with respect to A . **(C)** The average number of peaks $\bar{P}$ as a function of (G,A) for one run of the sorting algorithm on a WS-FHN network (*left*) and one run on the *β*C-FHN network (*right*) and for 20 equispaced values of *G* ∈ [0.15, 0.345]. As sortedness increases, the drive necessary for global, synchronised activation of the network decreases.

Figure 2 depicts a WS network (Figure 2A, top row) and a *β*C network (Figure 2B, top row) at five iterations of the sorting algorithm. The WS network can be described by a set of nodes equispaced along a ring, and when the rewiring probability is small, the majority of connections are between nodes that are close to one another on the ring. For our choice of *D* = 12 (and *β* = 0.2), the majority of connections will occur between an arbitrary node and the *D*/2 = 6 closest nodes in either direction. Figure 2A shows that as A increases, the population 1 nodes (blue points) begin to form clusters along the ring due to the high interconnectivity amongst near neighbours. They do not necessarily form a single cluster at convergence (*a* = 314 in this example), but due to the rewirings, many connections between clusters exist (blue lines). In the case of a *β*C network (Figure 2B), we see the emergence of localised population 1 clusters culminating in a single cluster at convergence (*a* = 239 in this example).

#### 2.4.2 Node selection probabilities

In this section, we formulate the node selection pdf used in the network sortedness adjustment algorithm. We assume that the selection of node from *P*_1_ is independent of the selection of node from *P*_2_:

$$f\left(i,j\right)=f_{P_{1}}\left(i\right)f_{P_{2}}\left(j\right),\;i\in P_{1},j\in P_{2}.$$ (19)

One choice would set *f*_1_ and *f*_2_ to be uniform over *P*_1_ and *P*_2_, respectively. This is the choice we take for the WS networks.

Empirical observations of the algorithm applied to the *β*C networks, however, demonstrate that clusters of population 1 nodes tend to form at the edge of the domain. As discussed in Section 2.3.2, we wish to avoid this scenario. The tendency for clusters to form near the edge occurs because of the spherical nature of our lattice domain. In particular, a uniform choice for *f*_1_ and *f*_2_ means that nodes at the centre of the domain are less likely to be selected under a uniformly random sampling of indices than those at the edge because the number of nodes in the network increases superlinearly with respect to the domain radius. Therefore, we derive choices for $f_{P_{k}}$ that equalise the probability of a node being selected on the basis of its radial coordinate. The heuristic for generating $f_{P_{1}}$ will be the same as that for generating $f_{P_{2}}$ up to the population identity.

Denote the radial distance from the origin of node $i\in N_{N}$ by $r_{i}={\left(x_{i}^{2}+y_{i}^{2}+z_{i}^{2}\right)}^{1/2}\in R_{\geq 0}$ where $\left(x_{i},y_{i},z_{i}\right)\in R^{3}$ are the Cartesian coordinates of the location of the node. We define a sequence of intervals, $I_{n}=\left[\left(n-1\right)\delta r,\;n\delta r\right]$ , for *n* = 1, … 8 with *δr* = *r*_max_/8 where *r*_max_ = max_*i*_{*r*_*i*_} so that each node is assigned to exactly one interval. The set of nodes from *P*_*k*_ belonging to a given interval $I_{n}$ is given by $R_{n,P_{k}}=\left\{i\in N_{N}|r_{i}\in I_{n},\;i\in P_{k}\right\}$ . Using these set definitions, the pdf $f_{P_{k}}$ may be defined as

$$f_{P_{k}}\left(i\right)=\frac{1}{Q|R_{n_{i},P_{k}}|},\;i\in N_{N}.$$ (20)

where $R_{n_{i},P_{k}}$ is such that $r_{i}\in I_{n_{i}}$ and *Q* is a normalisation factor ensuring that $\sum_{i\in P_{k}}f_{P_{k}}\left(i\right)=1$ . This choice for $f_{P_{k}}$ reweights the probability of a given node being selected by a factor proportional to the number of nodes from the same population within a spherical annulus with inner and outer radii specified by the boundaries of the intervals $I_{n}$ . This reweighting favours selecting nodes closer to the centre of the domain over those more distal.

Pseudocode for the *β*C network generation and network sortedness manipulation algorithm is provided in the Supplementary Section S2.

### 2.5 Evaluation of collective dynamics

To characterise the network dynamics, we consider two features based on the Ca^2+^ trajectories across all nodes, namely, the mean number of peaks $\left(\bar{P}\right)$ and the time-averaged degree of *phase* synchronisation $\left(\bar{R}\right)$ calculated as the average magnitude of the Kuramoto order parameter (see Supplementary Section S3). The mean number of Ca^2+^ peaks across all nodes is proportional to the *network participation*, that is, the fraction of nodes that undergo oscillation. The value of $\bar{R}$ captures the *network coordination*, tracking how closely the phases of the Ca^2+^ trajectories stay to one another across the simulation duration. We additionally define ${\bar{P}}_{k}$ and ${\bar{R}}_{k}$ where *k* ∈ {1, 2} to be the mean number of peaks in Ca^2+^ and the time-averaged degree of phase synchronisation across nodes in population *k*, respectively.

## 3 Results

### 3.1 For strong coupling, increasing sortedness decreases the necessary drive for a phase transition from quiescence into global and synchronised activity

We ran the sorting algorithm to convergence once on a typical WS network and again on the *β*C network to produce the sets $P_{k}$ for both network types (denote them WS- $P_{k}$ and *β*C- $P_{k}$ for *k* ∈ {1, 2}). For the WS network, this means specifically that we ran the Watts-Strogatz model of graph generation once to create a single small-world graph defining the connectivity structure of the network. We then uniform-randomly selected 10% of the nodes to be in population 1, thus defining the population sets $P_{k}^{0}$ . These were used as the initial configuration for the sorting algorithm, which was run to convergence (Figure 2A, top row) producing WS- $P_{k}$ . For the *β*C network, the connectivity structure is fixed. We defined $P_{k}^{0}$ as above and ran the sorting algorithm to convergence (Figure 2B, top row) producing *β*C- $P_{k}$ . For both network structures (WS and *β*C), we then simulated the FHN dynamics on the network models defined by $P_{k}^{a}$ for all *a* ∈ [0, *a*_*final*_] over 20 equispaced values of *G* ∈ [0.15, 0.345] with strong coupling $\left({\bar{g}}_{\mathrm{coup}}=0.1\right)$ . In this case, we used the same set of initial conditions across runs.

Figure 2C shows the average number of peaks $\bar{P}$ as a function of (*G*, A ). We found that as sortedness increases, the drive necessary to activate the networks decreases. Moreover, we found that for strong coupling, this phase transition results in a transition from quiescence to global, synchronised activity ( $\bar{R}$ not shown but takes a value $\bar{R}\approx 1$ when $\bar{P}\neq 0$ ). This was the case for both the WS-FHN (left panel) and *β*C-FHN networks (right panel). Figures 2A, B (bottom rows) shows the dynamics of *V* (for WS-FHN and *β*C-FHN resp.) averaged over population 1 and 2 using a fixed value of *G*. This illustrates the strong synchronisation between populations (for strong coupling) as well as the phase transition as a function of A alone.

We next verified that this observed relationship is robust to changes in $P_{k}$ and *Y* (0). To do this, we first calculated a Latin hypercube of pairs (*G*, *a*). For each point, we used a different seed network $P_{k}^{0}$ and ran the sorting algorithm to find $P_{k}^{a}$ . We then ran dynamics on the network model using a different set of initial conditions *Y* (0). Note that for the WS models, each seed network was based both on a different WS graph and a different initial choice of indices for the population 1 nodes. For the SRK model, we used *G* ∈ [0.2, 0.6], ${\bar{g}}_{\mathrm{coup}}=10$ (strong coupling), and 5000 (*G*, *a*) pairs. For the FHN model, we used *G* ∈ [0.15, 0.34], ${\bar{g}}_{\mathrm{coup}}=0.1$ , and 10,000 (*G*, *a*) pairs. We then calculated the features ( $\bar{P}$ and $\bar{R}$ ) for all the points (G,A) (where A is calculated from $P_{k}^{a}$ for each point) and used them to train a Gaussian process regression (GP) model (using the fitrgp function in MATLAB). We then estimated the features on a regular grid of points (G,A) and used that to generate heatmaps for the features. Supplementary Figure S2 shows the process of generating these heatmaps for $\bar{P}$ in the case of the WS-FHN network models.

**FIGURE 3:**
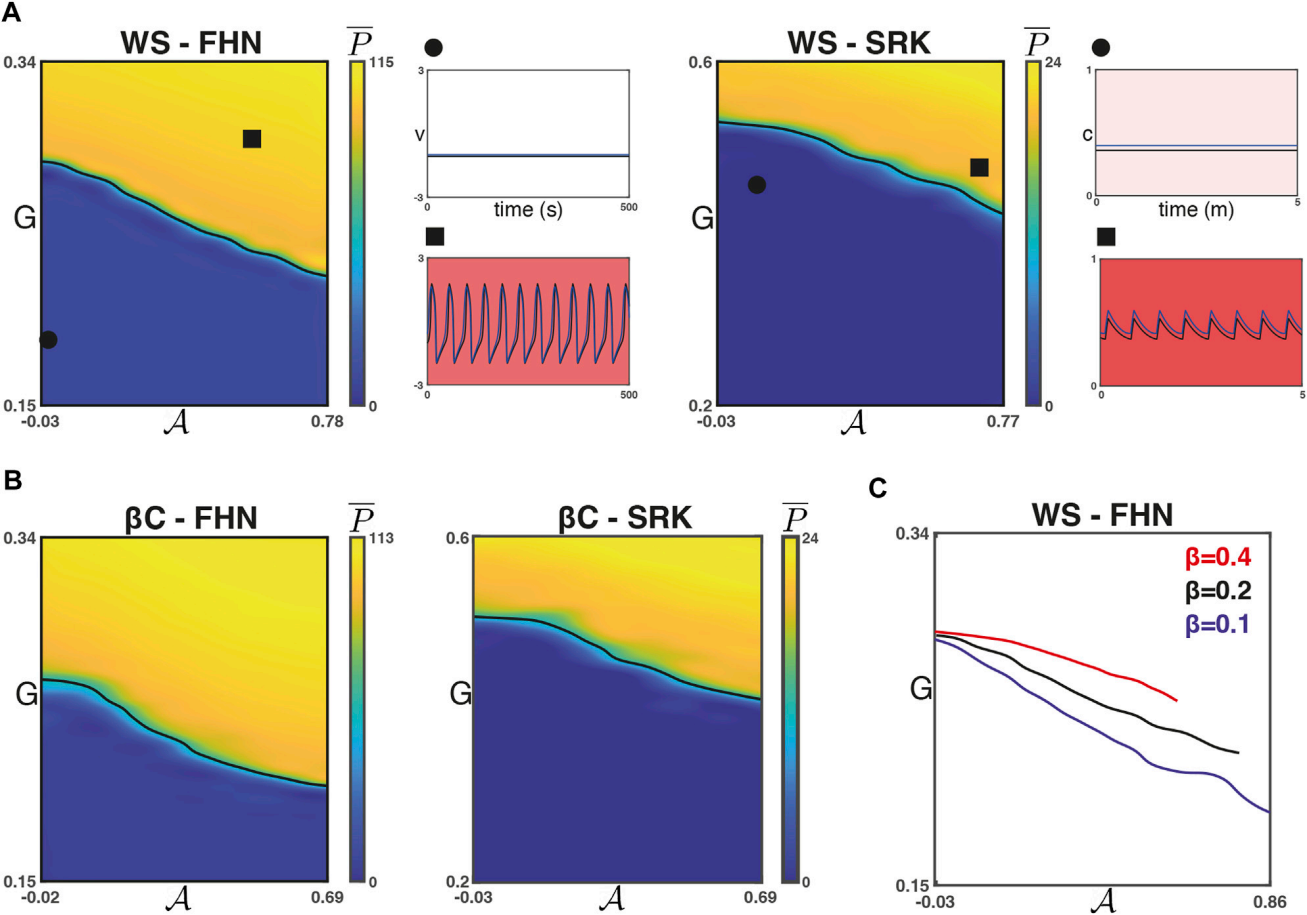
Phase transitions with respect to A for strong coupling. **(A)** The phase transition from quiescence to global, synchronised activity requires lower drive as sortedness increases in WS networks and for both models (FHN and SRK) when coupling is strong ( ${\bar{g}}_{\mathrm{coup}}=0.1$ for FHN and ${\bar{g}}_{\mathrm{coup}}=10$ for SRK). **(B)** This is likewise the case for the *β*C-FHN and *β*C-SRK model for strong coupling. **(C)** As *β* for the Watts-Strogatz model increases, the model transitions from a regular model with no rewiring to a random network. Here, we show the phase transition for three values of the rewiring probability. We found that the more regular a network, the lower the threshold *G* for the phase transition across the range of A .

We found that for each of the four combinations of network models, increasing A results in a decreased *G* required for a phase transition from quiescence to global, synchronised activity. Figure 3A shows $\bar{P}$ for the WS networks and Figure 3B shows the same for the *β*C networks. The synchronisation parameter $\bar{R}$ is again not shown but takes values $\bar{R}\approx 1$ when $\bar{P}\neq 0$ . For the heatmaps in Figures 3A, B, the $\bar{P}$ half-maximum value is shown as a black curve.

For the WS networks, the rewiring parameter *β* is related to the small-worldness of the network and inversely related to the regularity of the graph. Moreover, the maximum possible value of A will occur for *β* = 0, when population 1 nodes form a single cluster. We found that, in general, the sorting algorithm converges to larger values of A for smaller values of *β*. Figure 3C shows the $\bar{P}$ half-maximum value (using the Latin hypercube protocol described above for WS-FHN) for *β* ∈ {0.1, 0.2, 0.4}. We found that for a given value of A , the *G* value at the phase transition is smaller for smaller values of *β*. This supports the hypothesis that the networks with more localised connectivity, such as the WS graphs when *β* ≈ 0 and the *β*C graph, tend to have a lower activation threshold than networks with increased small-worldness (also compare Figures 3A, B). This is perhaps unsurprising because in the networks with localised connectivity, clusters of excitable nodes only interact with the less excitable nodes along some boundary. For example, in the *β*C network, the nodes in the centre of a population 1 cluster will be completely separate from population two nodes. Whereas in the networks with small-world properties (WS, *β* ≠ 0), very few population 1 nodes (if any) will have a node sortedness of 1.

### 3.2 Weak coupling and high sortedness leads to wave propagation in the FHN networks

Next, we studied the influence of coupling strength on the relationship between A , *G*, and the features of collective dynamics ( $\bar{P}$ and $\bar{R}$ ). To do this, we calculated a Latin hypercube of triplets $\left({\bar{g}}_{\mathrm{coup}},G,a\right)$ . As before, we used different seed networks to identify $P_{k}^{a}$ and ran the dynamics using different initial conditions *Y* (0). For the FHN model, we used *G* ∈ [0.15, 0.34], ${\bar{g}}_{\mathrm{coup}}\in \left[0.02,0.1\right]$ , and 20,000 $\left({\bar{g}}_{\mathrm{coup}},G,a\right)$ triplets. For the SRK model, we used *G* ∈ [0.2, 0.6], ${\bar{g}}_{\mathrm{coup}}\in \left[2,10\right]$ , and 10,000 triplets. We again calculated the features ( $\bar{P}$ and $\bar{R}$ ) for all the points $\left({\bar{g}}_{\mathrm{coup}},G,A\right)$ (where A is calculated from $P_{k}^{a}$ for each point) and used them train a GP model. We used these to draw isosurfaces (level sets) of $\bar{P}$ and $\bar{R}$ as functions of $\left({\bar{g}}_{\mathrm{coup}},G,A\right)$ .

**FIGURE 4:**
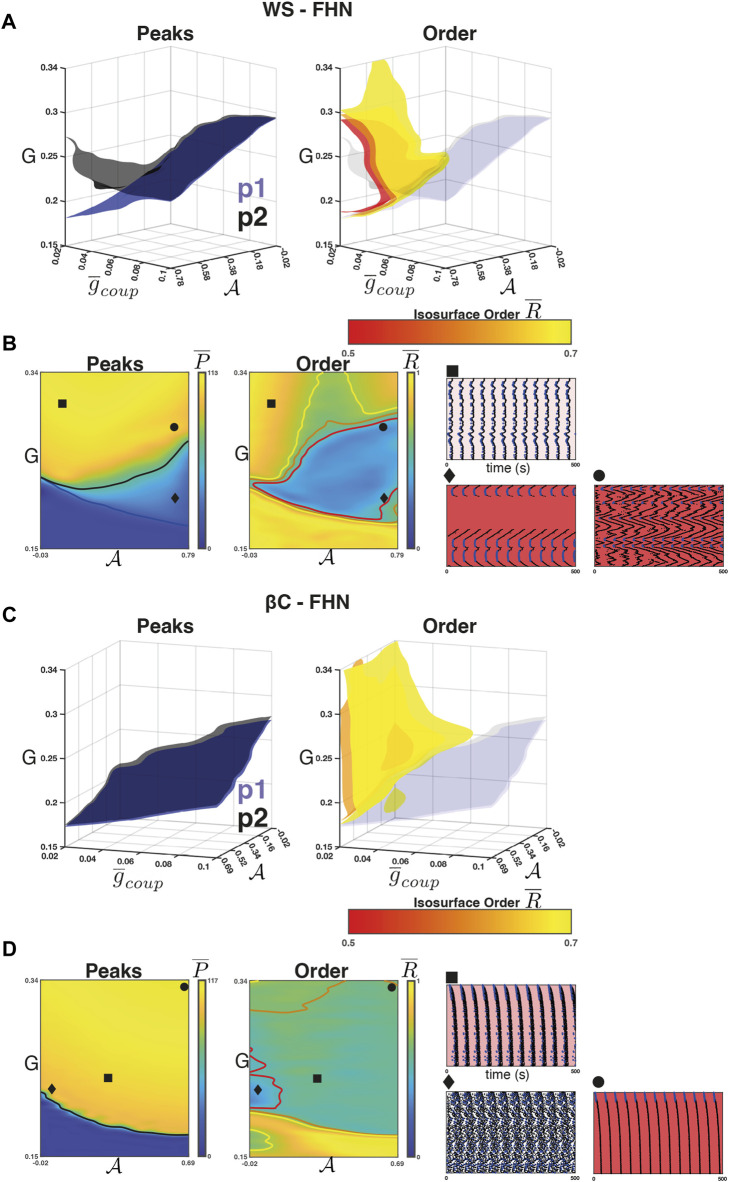
Weak coupling in the FHN networks. **(A)** *Left panel:* The isosurfaces for the ${\bar{P}}_{1}$ (blue) and ${\bar{P}}_{2}$ (black) half-maxima in the WS-FHN networks. Above these surfaces, population 1 and 2, respectively transition to population-wide activity. These surfaces separate for weak coupling and high sortedness, bounding a regime where population 1 is active but population 2 is only partially active. *Right panel:* The isosurfaces for $\bar{R}$ illustrate the boundary between low and high synchrony in the WS-FHN networks. For weak coupling and high sortedness, a region of low synchrony emerges. This region encompasses the region where population 1 is active and population 2 is partially active. **(B)** Heatmaps for $\bar{P}$ (*left panel*) and $\bar{R}$ (*right panel*) when ${\bar{g}}_{\mathrm{coup}}=0.02$ (weak). The intersection of each isosurface in **A** with the plane ${\bar{g}}_{\mathrm{coup}}=0.02$ is drawn as a contour line. Raster plots illustrate typical activity in the different regions bounded by these contours and nodes are arranged according to location along the ring. *Square* shows the synchronised activity when sortedness is low. *Diamond* shows the terminating waves that occur when sortedness is high but drive is low. *Circle* shows the non-terminating waves that occur for high sortedness and intermediate drive. **(C)** *Left panel:* The isosurfaces for the ${\bar{P}}_{1}$ (blue) and ${\bar{P}}_{2}$ (black) half-maxima in the *β*C-FHN networks. *Right panel:* The isosurfaces for $\bar{R}$ illustrate the boundary between low and high synchrony in the *β*C-FHN networks. Low synchrony emerges across sortedness and drive when coupling is weak. For low sortedness and when drive is very close to the transition between quiescence and activity [see **(D)** *Diamond*], there exists an region of low synchrony that persists for higher values of ${\bar{g}}_{\mathrm{coup}}$ (i.e., yellow bump). **(D)** Heatmaps for $\bar{P}$ (*left panel*) and $\bar{R}$ (*right panel*) when ${\bar{g}}_{\mathrm{coup}}=0.02$ (weak). For the raster plots, nodes are ordered according to radial distance from the centre of the lattice. *Square* shows the fast but wave-like propagation of activity through the lattice for intermediate drive and sortedness. *Diamond* shows the asynchronous activity for low sortedness and low drive. *Circle* shows fast wave-like propagation when sortedness and drive are high.

For the WS-FHN networks, we found that the intra-population isosurfaces (e.g., ${\bar{P}}_{1}$ and ${\bar{P}}_{2}$ ) corresponding to the phase transition between quiescence and activity separate for weak coupling and high A . Figure 4A (*left*) shows the isosurfaces $\left\{\left({\bar{g}}_{\mathrm{coup}},G,a\right)|{\bar{P}}_{k}=0.5*\max{\bar{P}}_{k}\right\}$ for *k* = 1 (blue, population 1) and *k* = 2 (black, population 2). For population 1, the phase transition threshold (*G*) decreases as A increases for all values of ${\bar{g}}_{\mathrm{coup}}\in \left[0.02,0.1\right]$ . However, for population 2, this relationship was only observed for strong coupling. For weak coupling and high sortedness, ${\bar{P}}_{2}$ is an increasing function of A . Figure 4B (*left*) shows the heatmap of $\bar{P}$ for ${\bar{g}}_{\mathrm{coup}}=0.02$ as well as the contour curves $\left\{\left({\bar{g}}_{\mathrm{coup}}=0.02,G,a\right)|{\bar{P}}_{k}=0.5*\max{\bar{P}}_{k}\right\}$ for *k* = 1 (blue, population 1) and *k* = 2 (black, population 2). In the region between these contours, we found a region where all of population 1 activates, but many population 2 nodes do not. Figure 4B (*diamond*) shows a raster plot of all nodes (population 1 in blue) arranged so that neighbours on the raster plot correspond to neighbours on the ring (see Figure 2A). Population 1 clusters become active and activate the population 2 nodes neighbouring the cluster boundaries. The population 2 nodes then display wave-like propagation (i.e., time-delayed) that terminates before all the nodes have become active. The waves occur because in the region, sortedness is sufficiently high that the primary signal activating many population 2 nodes comes *via* local connections from previously activated population 2 nodes. Moreover, the long-range connections from population 1 nodes are weak and insufficient to activate population 2 in a synchronous manner [as in Figure 4B (*square*)]. To show this, we reran the experiment using *β* = 0 (no rewirings), which is shown in Supplementary Figure S3. We found that regardless of choice of coupling strength and sortedness, if the network activates, it produces waves emanating from one or more clusters of population 1 nodes. Moreover, we did not observe wave termination when *β* = 0, implying that it is the rewiring of connections that leads to the wave termination. Since the activation of a population 2 node is dependent on the strength of local connections in the chain and rewiring decreases the number of local connections, there exist some population 2 nodes (or clusters) that do not activate, thus terminating the wave.

Figure 4A (*right*) shows the isosurfaces $\left\{\left({\bar{g}}_{\mathrm{coup}},G,a\right)|\bar{R}=\bar{r}\right\}$ for values between $\bar{r}=0.5$ (red) and $\bar{r}=0.7$ (yellow). These isosurfaces illustrate the boundary between low and high phase synchrony for the network as a whole. We found that for weak coupling and high A , a region of low synchrony emerges. Figure 4B (*right*) shows the heatmap of $\bar{R}$ for ${\bar{g}}_{\mathrm{coup}}=0.02$ and the contour curves corresponding to Figure 4A isosurfaces. This low synchrony is a result of the wave propagation that occurs in the region. Note that the region of low synchrony encapsulates the population 2 phase transition. There exists a region where population 2 is fully activated, but the synchrony is still low. Figure 4B (*circle*) shows an example where waves emit from the population 1 clusters, but the drive *G* to population 2 nodes is high enough to prevent rewiring-based termination of the waves. If *G* is increased further, synchrony re-emerges due to the fact that even weak (and few) long-range connections from population 1 can synchronise the primed population 2 nodes.

For the *β*C-FHN networks, we repeated the experiment as described above. In this case, however, the raster plots are arranged by radial distance from the centre of the lattice (Figure 4D *rasters*). Unlike in the case of WS-FHN networks, ${\bar{P}}_{1}$ and ${\bar{P}}_{2}$ did not separate for weak coupling. We found that across the range of ${\bar{g}}_{\mathrm{coup}}$ , the phase transition threshold is a decreasing function of A and that it represents a phase transition of the entire network [Figures 4C, D (*left*)]. When coupling is weak (Figure 4D shows ${\bar{g}}_{\mathrm{coup}}=0.02$ ), the synchrony of the network decreases within the entirety of the active region. This is due to the propagation of waves from population 1 clusters, similar to the case of WS-FHN with no rewirings, however, these waves propagate quickly due to the supralinearity in the number of nodes being recruited over time. Figure 4D (*square* and *circle*) show two examples where a cluster of population 1 nodes near the centre of the lattice emit such waves. Finally, we found a region for low A and low *G* (in the active region) where asynchronous activity was observed [Figure 4D (*diamond*)]. This is the only region where we observed asynchrony, and it corresponds to the region where a maximal value of ${\bar{g}}_{\mathrm{coup}}$ is necessary to synchronise the network (Figure 4C, see the bump in the yellow isosurface).

### 3.3 Weak coupling and high sortedness leads to resonance in the SRK networks

**FIGURE 5:**
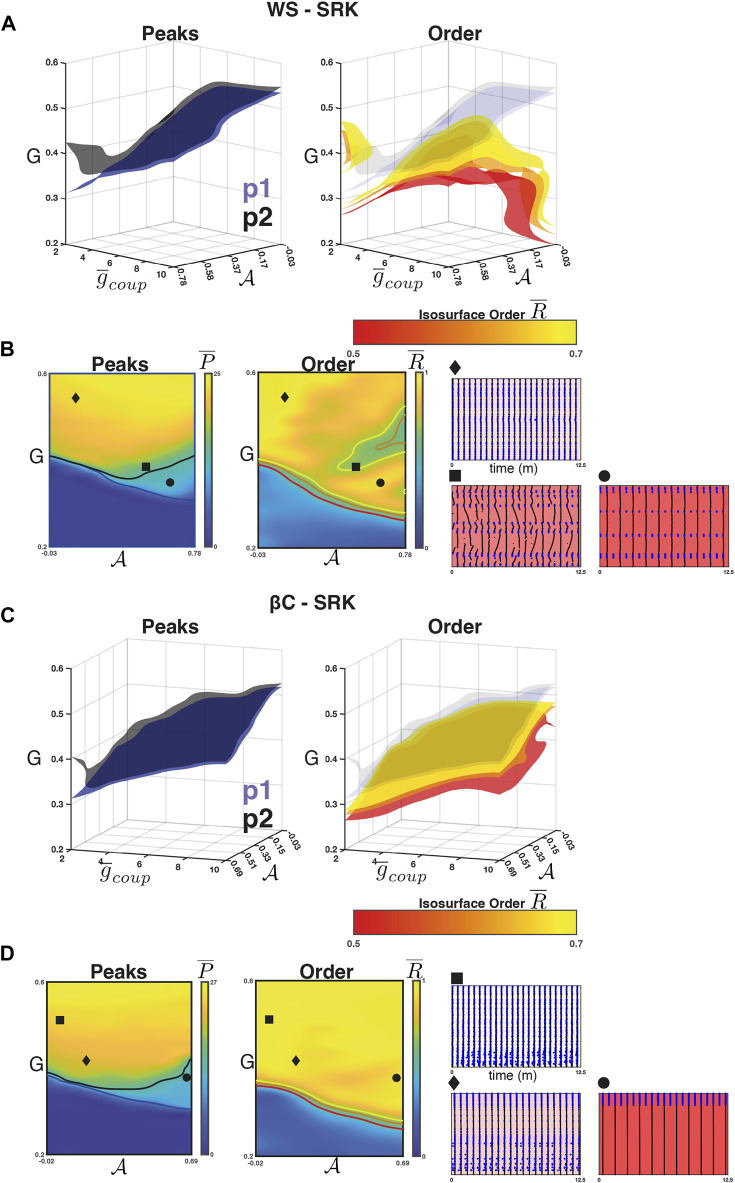
Weak coupling in the SRK networks. **(A)** *Left panel:* The isosurfaces for the ${\bar{P}}_{1}$ (blue) and ${\bar{P}}_{2}$ (black) half-maxima in the WS-SRK networks. Above the surfaces, population 1 and 2, respectively, transition to population-wide activity. These surfaces separate for weak coupling and high sortedness, revealing a 2:1 resonance regime. *Right panel:* The isosurfaces for $\bar{R}$ illustrate the boundary between low and high synchrony in the WS-SRK networks. For weak coupling and high sortedness, low synchrony emerges at the transition where population 2 becomes fully active. **(B)** Heatmaps for $\bar{P}$ (*left panel*) and $\bar{R}$ (*right panel*) when ${\bar{g}}_{\mathrm{coup}}=2$ (weak). The intersection of each isosurface in **A** with the plane ${\bar{g}}_{\mathrm{coup}}=2$ is drawn as a contour line. Raster plots illustrate the activity and nodes are arranged according to location along the ring. *Diamond* shows fully synchronous activity. *Circle* shows the 2:1 resonance. *Square* shows the irregular activity in the low synchrony region. **(C)** *Left panel:* The isosurfaces for the ${\bar{P}}_{1}$ (blue) and ${\bar{P}}_{2}$ (black) half-maxima in the *β*C-SRK networks. *Right panel:* The isosurfaces for $\bar{R}$ show that synchrony is high everywhere the network is active in the *β*C-SRK networks. **(D)** Heatmaps for $\bar{P}$ (*left panel*) and $\bar{R}$ (*right panel*) when ${\bar{g}}_{\mathrm{coup}}=2$ (weak). For the raster pots, nodes are ordered according to radial distance from the centre of the lattice. *Square* shows fully synchronous activity for low sortedness. *Diamond* shows fully synchronous activity for intermediate sortedness. *Circle* shows the 2:1 resonance.

For the WS-SRK networks, we found that the intra-population isosurfaces corresponding to the phase transition between quiescence and activity separate for weak coupling and high A (Figure 5A *left panel*). The population 1 phase transition threshold (*G*) decreases as A increases for all values of ${\bar{g}}_{\mathrm{coup}}\in \left[2,10\right]$ . For population 2, this relationship is observed for strong coupling, but for weak coupling and high sortedness, ${\bar{P}}_{2}$ is an increasing function of A . Figure 5B (*left*) shows the heatmap of $\bar{P}$ for ${\bar{g}}_{\mathrm{coup}}=2$ as well as the contour curves $\left\{\left({\bar{g}}_{\mathrm{coup}}=2,G,a\right)|{\bar{P}}_{k}=0.5*\max{\bar{P}}_{k}\right\}$ for *k* = 1 (blue, population 1) and *k* = 2 (black, population 2). Within this region, we observe a region of 2:1 resonance, wherein population 1 is active and synchronised, whilst population 2 is active and synchronised but with half the frequency of population 1 (Figure 5B *circle*). Nodes in the SRK model produce bursts of electrical activity resulting in the slow build-up of Ca^2+^ (i.e., *c*). As Ca^2+^ concentration increases, hyperpolarising K^+^ channels open and terminate the burst. During the inactive period, Ca^2+^ concentration decreases and the cell becomes increasingly excitable over time. At this level of coupling, the population 2 nodes are still capable of being driven to activity by nodes in population 1. However, they also require a longer recovery time (interburst interval) leading to the observed resonance. It is worth noting that wave-like activity was not generally observed, due to the fact that our variable of interest, *c*, is very slow with respect to the coupled variable, *V* (see Figure 1). Thus, the time delays in activation between nodes occur on the order of oscillations in *V* (i.e., action potentials), which are very short relative to the oscillation frequency of *c* (i.e., bursts). This is illustrated in Supplementary Figure S4, which shows the behaviour of the WS-SRK networks when *β* = 0. For strong coupling, the activity of the network is still nearly synchronous despite only having local connections. For weak coupling, we did observe waves (and lowered synchrony), however, they are fast relative to the period of *c* dynamics (compare Supplementary Figure S4B *triangle* to Supplementary Figure S3B *circle*). Interestingly, as in the case of WS-FHN networks, we did not observe the separation in ${\bar{P}}_{1}$ and ${\bar{P}}_{2}$ when *β* = 0 and there was no 2:1 resonance region. We conclude, therefore, that it is the rewiring that leads to the emergence of 2:1 resonance. The lowered number of local connections in the chain at the boundaries between population 1 and population 2 clusters do have the effect of terminating a “wave” (where synchronised activity is the limit as wave speed tends to infinity), as in the case of the WS-FHN networks, however, it only occurs half the time because of the slow recovery dynamics of the SRK model. The tradeoff is that the increased number of rewiring connections allows synchronisation in the 2:1 resonance region (compare Figure 5B *circle* to Supplementary Figure S4B *triangle*).

Figure 5A (*right*) shows the isosurfaces $\left\{\left({\bar{g}}_{\mathrm{coup}},G,a\right)|\bar{R}=\bar{r}\right\}$ for values between $\bar{r}=0.5$ (red) and $\bar{r}=0.7$ (yellow). These isosurfaces show that the region of lowered phase synchrony does not fully encompass the 2:1 resonance regime. Rather, it surrounds the ${\bar{P}}_{2}$ isosurface for weak coupling and high sortedness. Figure 5B (*right*) shows the heatmap of $\bar{R}$ for ${\bar{g}}_{\mathrm{coup}}=2$ and the contour curves corresponding to Figure 5A isosurfaces. This region of lowered synchrony corresponds to the transition between the 2:1 resonance region (Figure 5B *circle*) and the fully synchronised region (Figure 5B *diamond*). Within this region, we found a region with irregular activity (Figure 5B *square*) with a mixture of network-wide, synchronous bursts and wave-like activity. This is due to the variability in local and non-local connections among the population 2 nodes interacting with the slow recovery of Ca^2+^ very near the population 2 phase transition. Consider the raster plot shown in Figure 5B (*square*) and note that the population 1 nodes fire regular, synchronous bursts (beats). With a 3:1 resonance, the local and global connections together are strong enough to elicit a (roughly) synchronous burst. On the other beats, the small number of global connections are too weak to induce synchrony (as the population 2 Ca^2+^ concentration is still recovering) and instead wave propagation or termination occurs.

For the *β*C-SRK networks, we repeated the experiment and found that a 2:1 resonance regime emerged in this case as well. Figures 5C, D (*left panel*) shows the separation in ${\bar{P}}_{1}$ and ${\bar{P}}_{2}$ for weak coupling and high A , and Figure 5D (*circle*) shows an example raster plot. Unlike in the case of the WS-SRK networks, synchrony is strong everywhere, including the 2:1 resonance region and the transition to the fully active region (Figures 5C, D *right panel*). We did find some examples of the irregular behaviour described above (notice the slight dark patch in Figure 5D *right panel* at the transition boundary), but this is less prominent in the *β*C-SRK networks, likely due to the regularity of the graph.

## 4 Discussion

In this manuscript, we demonstrated how transitions to globally-coordinated activity are dependent on the degree of sortedness in population excitability. We used two prototypical models of cellular excitability where a small population was highly excitable, whilst a larger population was less excitable. This scenario has recently been hypothesised to be relevant in beta cell networks, in particular in the description of “hubs,” populations displaying high functional connectivity (Johnston et al., 2016), ‘wave-initiators’ (also known as “leaders”) (Šterk et al., 2023), and “first-responders” (Kravets et al., 2022). As the global drive to the network was increased, activity across the network transitioned from a globally inactive state to one in which subsets of nodes became active and synchronised their activity. By perturbing the spatial distribution of the highly excitable population, we showed that the drive strength at which such transitions occur is dependent on the sortedness of the network. Moreover, we highlighted the presence of other types of network solution, including partially synchronised states, slow waves, and 2:1 resonant states and explored how these depended on sortedness. These results have implications for insulin secretion in the pancreatic islets of Langerhans, and more general implications regarding transitions to synchrony and other forms of collective dynamics in networks of coupled, excitable units.

Pulsatile insulin secretion is a key component of healthy islet function and is driven by the coordinated electrical activity of *β*-cell populations (Bertram et al., 2007). In turn, the coordination of electrical activity is facilitated by local cell-cell communication through the gap junctional network (Ravier et al., 2005). Recently, many studies have demonstrated the existence of *β*-cell heterogeneity and its importance in determining network dynamics (Stožer et al., 2013; Benninger et al., 2014; Johnston et al., 2016; Westacott et al., 2017; Salem et al., 2019; Benninger and Kravets, 2021; Nasteska et al., 2021; Kravets et al., 2022; Šterk et al., 2023). Thus, a key question in this work has been to study the potential impact of the cytoarchitecture (e.g., spatial layout of heterogeneity) in modulating these dynamics. Our work suggests that it may be key to fully understanding the impact of heterogeneity on collective dynamics. We chose to focus on one form of heterogeneity, the cell-intrinsic excitability, as a case study; however, it is likely that cytoarchitecture of *β*-cell networks will modulate the impact of heterogeneity more generally.

We focussed on heterogeneity in cell-intrinsic excitability because of its critical role in delineating subpopulations of pancreatic beta cell networks. High excitability is a feature of both the first-responder cells in the transient phase of glucose-stimulated insulin secretion (GSIS) (Salem et al., 2019; Benninger and Kravets, 2021; Kravets et al., 2022) and the wave-initiator population during the pulsatile phase of GSIS (Benninger et al., 2014). The existence of these populations has been heavily implicated as a feature of healthy pancreatic islets, as has their critical roles in driving the collective dynamics required for GSIS. However, defining these subpopulations is complex because they likely arise as an emergent property of the network, relying on an interplay of both cell-intrinsic properties (i.e., excitability and metabolism) and network properties (i.e., coupling strength and non-uniformity of heterogeneity) (Benninger et al., 2014; Westacott et al., 2017; Salem et al., 2019; Kravets et al., 2022; Šterk et al., 2023). For example, it appears that both first-responder cells (Kravets et al., 2022) and wave-initiator cells (Šterk et al., 2023) may have weaker local connectivity, a property that would isolate them from the hyperpolarising influence of less-excitable cells and could improve network responsiveness. Although our model did not include heterogeneous coupling; we did observe that weaker coupling lowers the activation threshold of the network (Figures 4A, 5A). Likewise, we demonstrated that the spatial aggregation of highly-excitable nodes, a network property previously demonstrated by Westacott et al. (2017), can also have this effect. On the other hand, our work also illustrates potential tradeoffs in these network features. When coupling is too weak, the network can fail to synchronise and waves can fail to propagate. When sortedness is too high, excitable clusters can fail to recruit the whole network.

A variety of simplifications have been used in modelling studies to provide insight into the collective dynamics of heterogeneous coupled oscillators. For example, in specific cases of heterogeneity under the assumption of isotropic coupling and in the limit of infinitely many nodes, the network state can be mapped exactly to a low dimensional description, in which boundaries representing dynamical transitions can be found in closed form (Watanabe and Strogatz, 1993; Ott and Antonsen, 2009; Montbrió et al., 2015). For networks with a large, but not infinite number of nodes, and with general types of structure and heterogeneity, such a transformation may not be available, meaning that exact prediction of transitions between dynamical states is largely intractable. Our approach was to perform Monte-Carlo sampling across networks and initial conditions to elucidate common behaviours and trends regarding such transitions, taking advantage of the robustness of results across trials. In spite of the analytical intractability of the system, our results are consistent with the notion that the dynamics of the full network could be projected to a low dimensional manifold (e.g., the manifold representing full synchronisation of the subpopulations). In this regard, there are a number of approaches under active development, such as polynomial chaos expansion (Ghanem and Red-Horse, 2017), dynamic mode decomposition (Kutz et al., 2016), and proper orthogonal decomposition (Berkooz et al., 1993), that may facilitate construction of an approximate low dimensional system that captures the core dynamical features of the full heterogeneous network, thus allowing for a deeper exploration of network state transitions in the future.

To perform our study, we developed an algorithm that perturbs the sortedness of the network in a directed manner. The algorithm can be adapted to a range of domain geometries and network architectures, as showcased in our studies of Watts–Strogatz networks. Although our study focussed on conditions in which there are only two different populations, Section 2 discusses how our metrics can be extended to networks with more population types. Moreover, while we focussed on the case where the cells were homogeneous within a subpopulation, small changes to the algorithm could allow for the study of multimodal parameter distributions. We hope that this work will facilitate further study into the interplay of node-intrinsic heterogeneity and network features in driving the function of excitable networks, in particular beta-cell networks. To this end, future work will focus on adapting the sortedness algorithm to multiple parameters, which would allow us to further interrogate the importance of coupling strength, metabolic activity, electrical excitability, and neighbourhood on the emergence of functional roles in heterogeneous, excitable networks.

## Data Availability

The datasets presented in this study can be found in online repositories. The names of the repository/repositories and accession number(s) can be found below: https://github.com/dgalvis/network_spatial.
