# Supplementary material for: Spatial distribution of heterogeneity as a modulator of collective dynamics in pancreatic beta-cell networks and beyond
Source: Front Netw Physiol. 2023 Mar 24;3:1170930. doi: 10.3389/fnetp.2023.1170930 (PMC7614376; doi:10.3389/fnetp.2023.1170930)
Supplement: Supplementary file 1 [file Image1.PDF]

Supplementary Material

S1 ADDITIONAL TABLES AND FIGURES REFERENCED IN THE MANUSCRIPT

Parameter	Value
a	0.7
b	0.8
τ	12.5
$\overline{g}_{coup}$	{varies}

Table S1. Parameter values of the FHN model.

Parameter	Value	Parameter	Value	Parameter	Value
C_m (fF)	5310	V_m (mV)	4	S_m (mV)	14
V_n (mV)	-15	S_n (mV)	5.6	κ_1 (mV)	65
κ_2 (mV)	20	$\overline{\tau}$ (ms)	37.5	$\overline{V}$ (mV)	-75
V_h (mV)	-10	S_h (mV)	-10	$\overline{g}_K$ (pS)	2500
$\overline{g}_{Ca}$ (pS)	1400	V_K (mV)	-75	V_{Ca} (mV)	110
K_d (μ M)	100	$\overline{g}_{K-Ca}$ (pS)	30000	f	0.001
k_{Ca} (ms^{-1})	0.03	α $\left(\frac{\mu\text{m}^3\text{Coul}}{\text{mMol}}\right)$	4.5061×10^{-6}	$\overline{g}_{coup}$ (pS)	{varies}

Table S2. Parameter values of the SRK model.

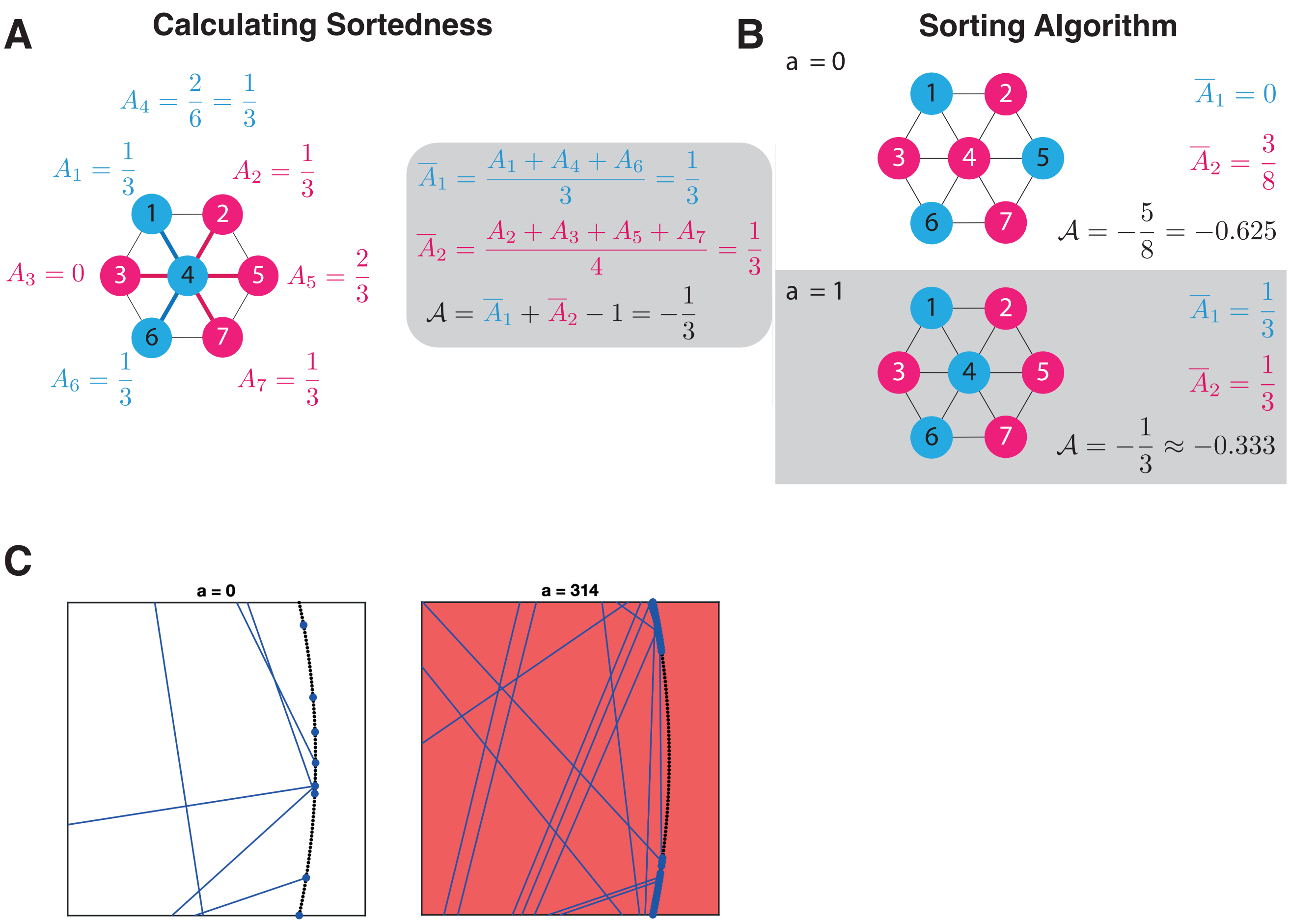

Figure S1. Sortedness and the sorting algorithm. A. An illustration of node, population, and network sortedness calculations on a small network. **B.** A successful iteration of the forward sorting algorithm. **C.** These are zoomed in portions of the WS networks, which are shown as black boxes in Fig. 2A. The initial network ($a = 0$) shows the population 1 nodes mostly isolated, but the final network ($a = 314$) shows two distinct clusters of several population 1 nodes.

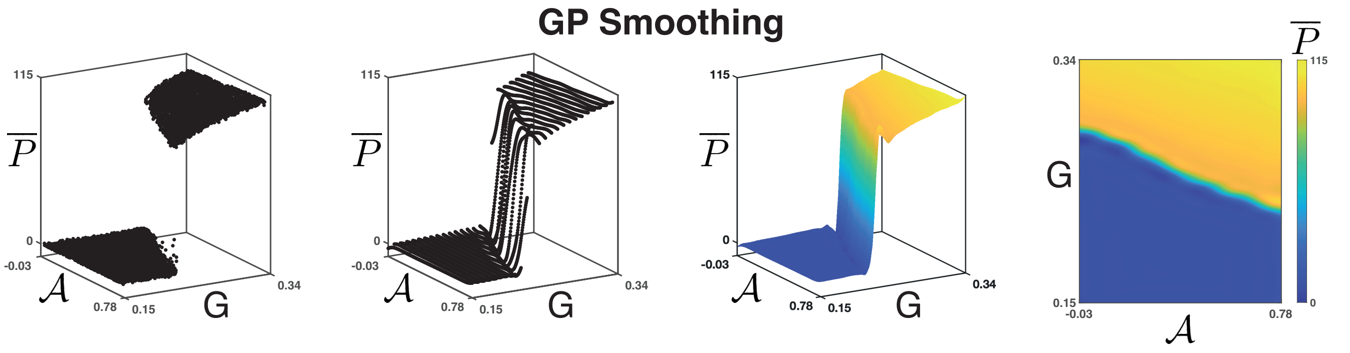

Figure S2. Gaussian process regression model. We now simulate dynamics on networks where (G, a) pairs are selected on a Latin hypercube. For each point, we use a different initial network P_k^0 and run the sorting algorithm to find the network P_k^a. We also use randomly selected initial conditions, $Y(0)$. In the case of the WS networks, we also use a different WS graph for each point. *Left panel* The feature $\overline{P}$ is shown as a scatter plot for each point $(G, \mathcal{A})$. *Middle-left panel* We use a Gaussian process regression model trained to the data on the Latin hypercube to estimate the points on a regular grid. *Middle-right panel* We can use this smoothed dataset to produce surface plots. *Right panel* We show the panels as heatmaps. Here, we show the resulting heatmap after training GP model with 10000 points using the WS-FHN network model. This is for the case where coupling is strong ($\overline{g}_{coup} = 0.1$).

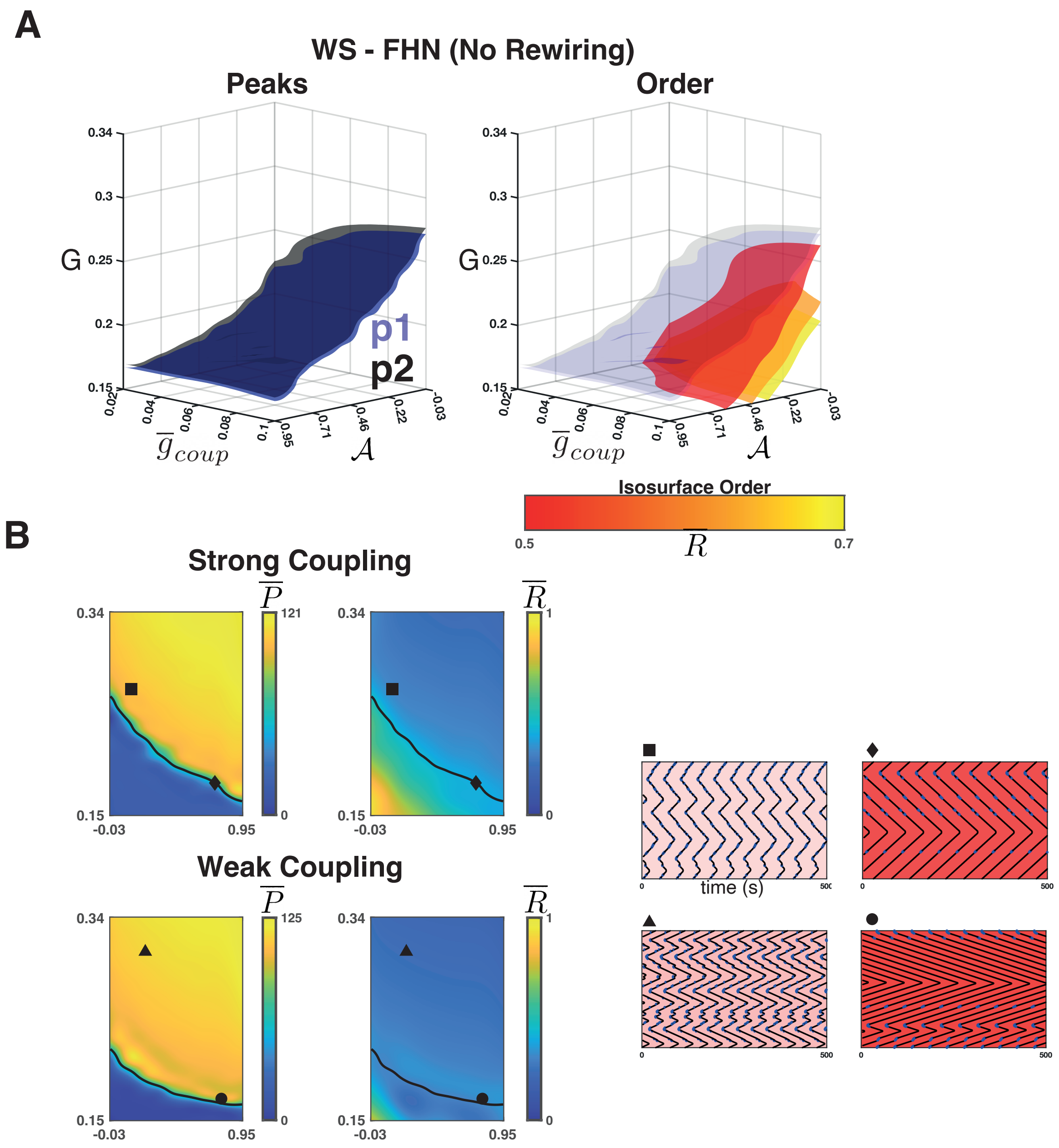

Figure S3. No rewiring in the WS-FHN networks. A. *Left panel:* The isosurfaces for the $\overline{P}_1$ (blue) and $\overline{P}_2$ (black) half-maxima in the WS-FHN networks with no rewiring. These surfaces do not separate. *Right panel:* The isosurfaces for $\overline{R}$ illustrate the boundary between low and high synchrony as well as its direction in the WS-FHN, $\beta = 0$ networks. Within the active region, synchrony is always low due to the propagation of waves. **B.** Heatmaps for $\overline{P}$ *(top left panel)* and $\overline{R}$ *(top right panel)* when $\overline{g}_{coup} = 0.1$ (strong). Heatmaps for $\overline{P}$ *(bottom left panel)* and $\overline{R}$ *(bottom right panel)* when $\overline{g}_{coup} = 0.02$ (weak). This shows that the phase transition is still a decreasing function of $\mathcal{A}$, but that synchrony is low regardless of coupling strength. *Square*, *diamond*, *triangle*, and *circle* show wave propagation for strong and weak coupling and low and high sortedness.

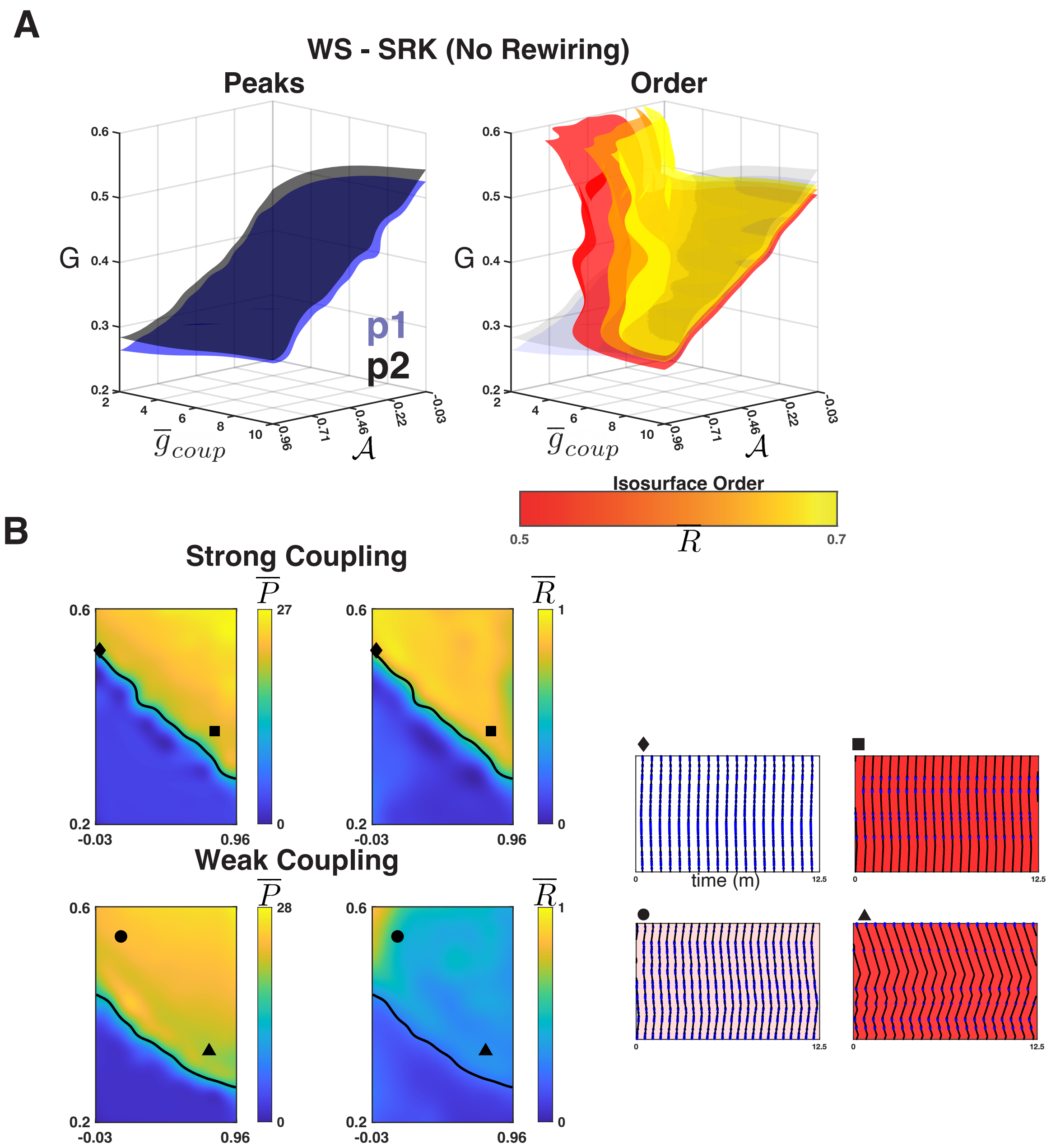

Figure S4. No rewiring in the WS-SRK networks. A. *Left panel:* The isosurfaces for the $\overline{P}_1$ (blue) and $\overline{P}_2$ (black) half-maxima in the WS-SRK networks with no rewiring. These surfaces do not separate. *Right panel:* The isosurfaces for $\overline{R}$ illustrate the boundary between low and high synchrony as well as its direction in the WS-SRK, $\beta = 0$ networks. Within the active region, synchrony is low for low to intermediate coupling due to wave propagation. **B.** Heatmaps for $\overline{P}$ *(top left panel)* and $\overline{R}$ *(top right panel)* when $\overline{g}_{coup} = 10$ (strong). Heatmaps for $\overline{P}$ *(bottom left panel)* and $\overline{R}$ *(bottom right panel)* when $\overline{g}_{coup} = 2$ (weak). This shows that the phase transition is still a decreasing function of $\mathcal{A}$, that synchrony is low for weak coupling, but that synchrony is still high for strong coupling. *Square* and *diamond* show synchronous activity for strong coupling. *Triangle*, and *circle* show wave propagation for weak coupling, but that the wave propagation remains fast relative to the period of c.

S2 DESCRIPTION OF THE ROUTINES USED TO GENERATE A βC LATTICE NETWORK AND PERFORM THE SORTING ALGORITHM ON IT

Algorithm 2-Algorithm 7 are used by Algorithm 1 which is described in the main text.

Algorithm 2 returns a set of points in $\mathbb{R}^3$ corresponding to the centres of spheres within a hexagonal close packed lattice (hcp). The input r_{ball} corresponds to the radius of the spheres within the lattice, which we set to $r_{ball} = 0.5$ so that the distance between any two nearest neighbors is $d_{ball} = 2r_{ball} = 1$. Algorithm 2 produces the hcp lattice using a sequence of scalings and shifts of a square lattice which takes the points $\{(x, y, z) \mid x, y, z \in \{1, \ldots, M\}\}$, where M is an integer corresponding to the number of spheres along the length of the lattice. We sought to embed a larger sphere, S_{net}, of radius R_{net} within the resulting hcp-lattice, and therefore, must choose M such that S_{net} is contained within the lattice. For the square lattice, a natural choice would be $M = 2R_{net}$, so that the length of the lattice equals the diameter of the sphere. However, for the hcp-lattice, the size of the resulting structure is $(M-1)x_{scale}+d_{ball} = Mx_{scale} = Md_{ball}$ by $(M-1)y_{scale}+d_{ball} > My_{scale}$ by $(M-1)z_{scale}+d_{ball} > Mz_{scale}$ (ignoring the shifts). To counteract this, we use:

$$M = \text{ceil}\left(\frac{2R_{net}}{\min([x_{scale}, y_{scale}, z_{scale}])}\right). \tag{S1}$$

We found that this choice of M generated a lattice which could fully embed the sphere, at least for our selection of $R_{net} = 5.55$ (in particular, we increased M and found that the number of nodes within the sphere did not increase).

Algorithm 3 first runs Algorithm 2 to produce an hcp-lattice. It then centres the lattice at the origin (i.e., at $(0, 0, 0)$) and finds all points that are within a sphere of radius R_{net} centred at the origin, which define the nodes in the network. It also returns N, the number of nodes in the spherical hcp-lattice ($N = 1,018$ in this work). Algorithm 4 establishes the Boolean adjacency matrix representing the connections between nodes in the spherical hcp-lattice. A connection exists between two nodes if they are at a distance of d_{ball} from one another. In other words, if two spheres (of radius r_{ball}) centred at the locations assigned to two nodes would be touching, then a connection exists between them. Algorithm 5 determines the population sets P_k for $k \in 1, 2$. It returns the number of nodes N_k in each population, the population membership sets, and the initial network sortedness value A_0. Algorithm 6 determines the selection probabilities for every pair of nodes ($\{(i, j) \mid i \in P_1, j \in P_2\}$). Algorithm 7 chooses a candidate swap, produces the population sets established by that swap, and calculates $\mathcal{A}$ for the updated population sets.

Algorithm 1 Algorithm for producing networks

Inputs:

N: number of nodes in network
a: number of iterations of swapping algorithm to attempt
Dir: signed integer determining whether algorithm runs forwards (positive) or backwards (negative)
ρ: proportion of population 1 nodes

Outputs:

$\mathcal{A}$: network sortedness value
P_1: population 1 set
P_2: population 2 set
n: number of swaps performed

```
function GenerateNetwork(N, a, Dir, ρ)
    (x, y, z), r, K ← EstablishLattice(N)
    N1, N2, P1, P2, 𝒜 ← AssignInitialPopulations(N, ρ)
    Term ← false                ▷ Boolean determining whether terminal network state has been reached
    n ← 0
    while (n < a) and (Term = false) do
        f, F, Q ← ComputeSelectionProbabilities(r[], N1, N2, P1, P2)
        m ← 0
        swap ← true             ▷ Boolean determining whether to attempt swaps
        while (m < N1 × N2) and (swap = true) do
            P̃1, P̃2, 𝒜p, k ← NodeSwap(f, F, Q, Dir, N1, N2, P1, P2)
            if sgn(𝒜p − 𝒜) = sgn(Dir) then
                P1, P2 ← P̃1, P̃2
                𝒜 ← 𝒜p
                n ← n + 1
                swap ← false
            else                ▷ Reject swap if 𝒜 does not change in the desired direction
                for l ← k to N1 × N2 do
                    F[l] ← F[l] − f[k]
                end for
                Q ← Q − f[k]
                m ← m + 1
            end if
        end while
        if swap = true then
            Term ← true         ▷ Terminal state has been reached
        end if
    end while
    return 𝒜, P1, P2, n
end function
```

Algorithm 2 Initialising HCP lattice

Inputs:

R_{net}: radius of the spherical lattice
r_{ball}: radius of balls around points in the lattice

Outputs:

$(x_1, y_1, z_1), \dots, (x_N, y_N, z_N)$: (x, y, z) coordinates of nodes
N: number of nodes in hcp-lattice

```
function EstablishHCPLattice(R_{net}, r_{ball})
    d_{ball} ← 2r_{ball}
    x_{scale} ← d_{ball}
    y_{scale} ← \sqrt{d_{ball}^2 - r_{ball}^2}
    z_{scale} ← \sqrt{\frac{2}{3}} d_{ball}
    x_{shift} ← r_{ball}
    y_{shift} ← -\frac{d_{ball}}{\sqrt{3}}
    M ← ceil(\frac{2R_{net}}{min([x_{scale}, y_{scale}, z_{scale}])})
    counter ← 0
    for i ← 1 to M do
        for j ← 1 to M do
            for k ← 1 to M do
                counter ← counter + 1
                x[counter] ← k × x_{scale}
                y[counter] ← j × y_{scale}
                z[counter] ← i × z_{scale}
                if j even then
                    x[counter] ← x[counter] + x_{shift}
                end if
                if i even then
                    y[counter] ← y[counter] + y_{shift}
                end if
            end for
        end for
    end for
    N ← M^3                                  ▷ Total number of nodes in the lattice
    return (x, y, z), N
end function
```

Algorithm 3 Initialising Sphere lattice

Inputs:

R_{net}: radius of the spherical lattice
r_{ball}: radius of points in the lattice

Outputs:

$(x_1, y_1, z_1), \ldots, (x_{N_{net}}, y_{N_{net}}, z_{N_{net}})$: $(x_{sphere}, y_{sphere}, z_{sphere})$ coordinates of nodes
$r_1, \ldots, r_{N_{net}}$: r_{sphere} radii of nodes
N_{net} number of nodes in the spherical lattice

```
function EstablishSphereLattice(R_net, r_ball)
    (x, y, z), N ← EstablishHCPLattice(R_net, r_ball)
    x ← x − mean(x)                              ▷ demean vector x
    y ← y − mean(y)                              ▷ demean vector y
    z ← z − mean(z)                              ▷ demean vector z
    r ← sqrt(x^2 + y^2 + z^2)                    ▷ compute norm over all points
    counter ← 0
    for i ← 1 to N do
        if r[i] <= R_net then                    ▷ Find members of hcp-lattice within sphere radius R_net
            counter ← counter + 1
            x_sphere[counter] ← x[i]
            y_sphere[counter] ← y[i]
            z_sphere[counter] ← z[i]
            r_sphere[counter] ← r[i]
        end if
    end for
    N_net ← counter                              ▷ Define number of nodes within the spherical domain
    return (x_sphere, y_sphere, z_sphere), r_sphere, N_net
end function
```

Algorithm 4 Initialising lattice

Inputs:

R_{net}: radius of the spherical lattice
r_{ball}: radius of points in the lattice

Outputs:

$(x_1, y_1, z_1), \dots, (x_N, y_N, z_N)$: (x, y, z) coordinates of nodes
$r_1, \dots, r_N$: r radial coordinate of nodes
$K \in \mathbb{R}^N \times \mathbb{R}^N$: connectivity matrix

```
function ESTABLISHLATTICE(R_net, r_ball)
    (x, y, z), r, N ← ESTABLISHSPHERELATTICE(R_net, r_ball)
    d_ball ← 2 r_ball
    for i ← 1 to N do
        for j ← 1 to N do
            dist ← sqrt((x[i] − x[j])^2 + (y[i] − y[j])^2 + (z[i] − z[j])^2)
            if dist = d_ball then
                K[i][j] ← 1
            else
                K[i][j] ← 0
            end if
        end for
    end for
    return (x, y, z), r, K
end function
```

Algorithm 5 Initialising populations

Inputs:

N: number of nodes in network
ρ: proportion of population 1 nodes

Outputs:

N_1, N_2: number of nodes in the respective population 1
P_1, P_2: population sets
$\mathcal{A}$: network sortedness

function ASSIGNINITIALPOPULATIONS(N, ρ)
    $U \leftarrow$ random permutation of $\{1, \ldots, N\}$
    $N_1 \leftarrow$ floor(ρN)
    $N_2 \leftarrow N - N_1$
    $P_1, P_2 \leftarrow$ integer array of length N_1, integer array of length N_2
    for $k \leftarrow 1$ to N_1 **do**
        $P_1[k] = U[k]$ ▷ Assign first N_1 elements of U to P_1
    end for
    for $k \leftarrow 1$ to N_2 **do**
        $P_2[k] = U[N_1 + k]$ ▷ Assign last N_2 elements of U to P_2
    end for
    $\mathcal{A} \leftarrow$ network sortedness value (17) using P_1 and P_2
    return N_1, N_2, P_1, P_2, $\mathcal{A}$
end function

Algorithm 6 Defining node pair selection probabilities

Inputs:

$r_1, \ldots, r_N$: radial coordinates of nodes
N_1, N_2: number of nodes in the respective population
P_1, P_2: population sets

Outputs:

$f \propto$ probability density function for node pair selection
$F \propto$ cumulative density function for node pair selection
Q: normalisation constant for f

```
function ComputeSelectionProbabilities(r[], N_1, N_2, P_1, P_2)
    f ← array of length N_1 × N_2,
    F ← array of length N_1 × N_2 + 1
    F[1] ← 0
    k, Q ← 0
    for i ← 1 to N_1 do
        for j ← 1 to N_2 do
            k ← k + 1
            p ← 1/R_{n_i,P_1} × 1/R_{n_j,P_2}      ▷ Weight probability of node pair being selected using (20)
            f[k] = p
            Q ← Q + p
            F[k] ← Q
        end for
    end for
    return f, F, Q
end function
```

Algorithm 7 Node population swapping

Inputs:

$f \propto$ probability density function for node pair selection
$F \propto$ cumulative density function for node pair selection
Q: normalisation constant for f
P_1, P_2: sets of indices of nodes in the respective population

Outputs:

$\widetilde{P}_1$, $\widetilde{P}_2$: population sets following node population swap
$\mathcal{A}_p$: network sortedness of network with node populations swapped
k: index of node pair swapped

function NODESWAP(f, F, Q, N_1, N_2, P_1, P_2)
  $u \leftarrow U(0, 1)$ ▷ Sample from unit uniform distribution
  $k \leftarrow 1$
  while $u < F(k)/Q$ **do**
    $k \leftarrow k + 1$
  end while
  $i, j \leftarrow k/N_2, (k-1) \bmod N_2 + 1$ ▷ Indices of selected population nodes
  $\widetilde{P}_1, \widetilde{P}_2 \leftarrow P_1, P_2$ ▷ Create copies of P_1 and P_2
  $\widetilde{P}_1(i), \widetilde{P}_2(j) \leftarrow P_2(j), P_1(i)$ ▷ Trial node population swap
  $\mathcal{A}_p \leftarrow$ network sortedness value (17) using $\widetilde{P}_1$ and $\widetilde{P}_2$
  return $\widetilde{P}_1$, $\widetilde{P}_2$, $\mathcal{A}_p$, k
end function

S3 EVALUATION OF COLLECTIVE DYNAMICS

For each node, the number of peaks was identified by searching for maxima with a peak prominence of $0.02\ \mu M$ in the Ca^{2+} timecourse (SRK) or 2 a.u. in the v timecourse (FHN) across the simulation duration.

For a network with N nodes, the time-dependent Kuramoto order parameter is a complex-valued scalar defined as

$$z(t) = R(t)\mathrm{e}^{i\Theta(t)} = \frac{1}{N}\sum_{j=1}^{N}\mathrm{e}^{i\theta_j(t)}, \tag{S2}$$

where $\theta_j(t)$ is the phase of the jth node, as extracted via a mean-subtracted Hilbert transform of the Ca^{2+} signal (SRK) or v signal (FHN) for node j. The argument of z, Θ, is the mean phase of the network whilst its magnitude, R, measures the degree of synchrony across the network. We sample the Ca^{2+} at equispaced time points $t_i = i\delta t$, $i = 0, \ldots T-1$ and record the time-averaged degree of synchronisation: $\overline{R} = \frac{1}{T}\sum_{i=0}^{T-1} R(t_i)$.
