# Supplementary material for: Spatial distribution of heterogeneity as a modulator of collective dynamics in pancreatic beta-cell networks and beyond
Source: Front Netw Physiol. Author manuscript; Available in PMC 2023 Mar 27. (PMC7614376; doi:10.3389/fnetp.2023.1170930)
Supplement: Supplementary file 1 [file EMS172422-supplement-SI.pdf]

S1 SUPPLEMENTAL MATERIAL

S1.1 Additional tables and figures referenced in the manuscript

Parameter	Value
a	0.7
b	0.8
τ	12.5
$\overline{g}_{coup}$	{varies}

Table S1. Parameter values of the FHN model.

Parameter	Value	Parameter	Value	Parameter	Value
C_m (fF)	5310	V_m (mV)	4	S_m (mV)	14
V_n (mV)	-15	S_n (mV)	5.6	κ_1 (mV)	65
κ_2 (mV)	20	$\overline{\tau}$ (ms)	37.5	$\overline{V}$ (mV)	-75
V_h (mV)	-10	S_h (mV)	-10	$\overline{g}_K$ (pS)	2500
$\overline{g}_{Ca}$ (pS)	1400	V_K (mV)	-75	V_{Ca} (mV)	110
K_d (μ M)	100	$\overline{g}_{K-Ca}$ (pS)	30000	f	0.001
k_{Ca} (ms^{-1})	0.03	$\alpha \left(\frac{\mu\text{m}^3\text{Coul}}{\text{mMol}}\right)$	4.5061×10^{-6}	$\overline{g}_{coup}$ (pS)	{varies}

Table S2. Parameter values of the SRK model.

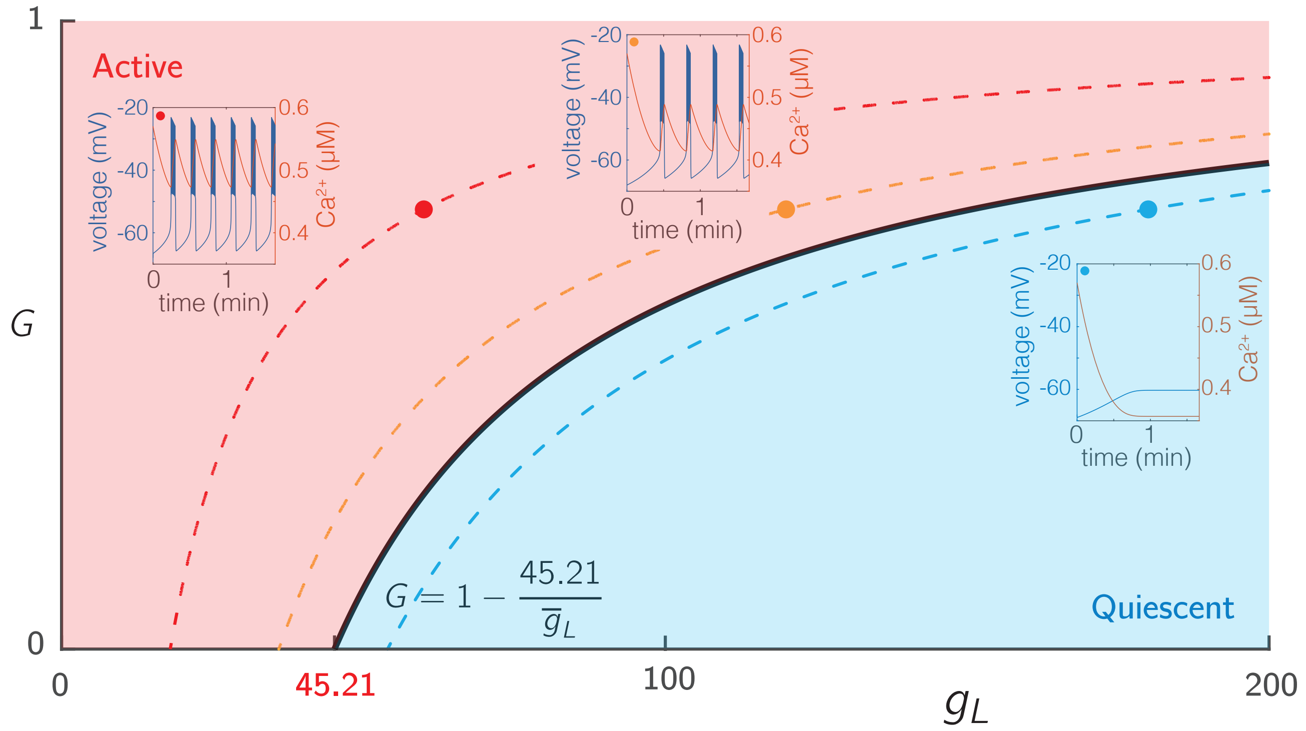

Figure S1. Excitability of single SRK cells. The voltage traces from three cells with varying levels of intrinsic excitability ($\overline{g}_L$), but the same level of drive ($G = 0.7$). The red, blue, and black traces show decreasing levels of excitability with values of $\overline{g}_L = 60$, 120, and 180, respectively. More excitable cells have a shorter interburst interval. The solid black line represents a Hopf bifurcation as a function of both G and g_L. At the lowest drive ($G = 0$), the Hopf bifurcation occurs for $\overline{g}_L = 45.21$ pS. The dotted lines represent "level sets" of the $(\overline{g}_L, G)$ parameter space, along which the behaviour of the single cell is identical. Data for the bifurcation diagram was computed using XPP 8.0 [1].

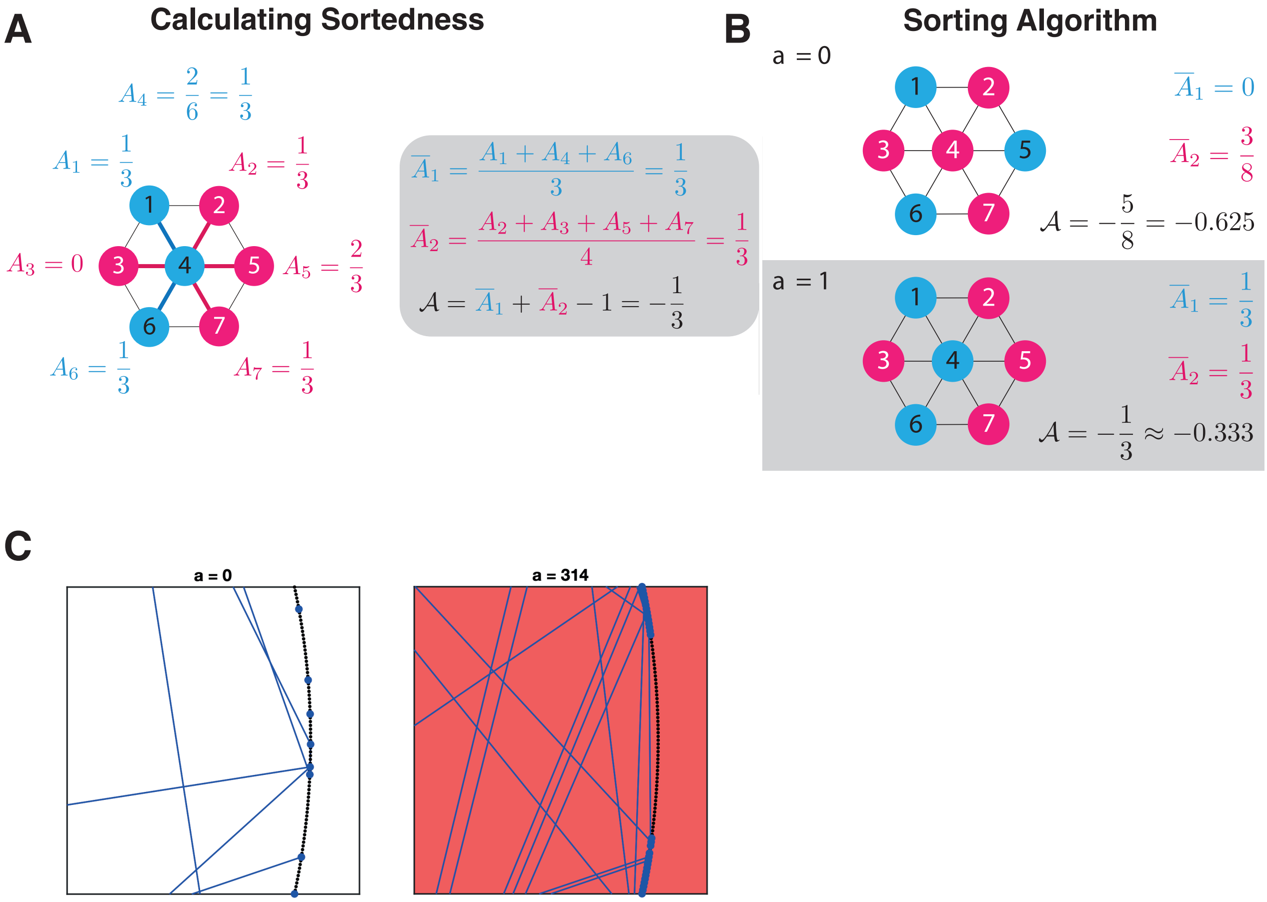

Figure S2. Sortedness and the sorting algorithm. A. An illustration of node, population, and network sortedness calculations on a small network. **B.** A successful iteration of the forward sorting algorithm. **C.** These are zoomed in portions of the WS networks, which are shown as black boxes in Fig. 2**Dynamics on networks defined by the sorting algorithm. A.** *Top Row:* Five iterations of the sorting algorithm on a WS graph (with $\beta = 0.2, D = 12$). The majority of connections in this graph ($\approx 80\%$) are between a node and its $D/2 = 6$ closest neighbours in each direction along the ring. The other $\approx 20\%$ of connections are randomly selected for rewiring. In each panel, only the connections within population 1 (blue lines) are shown, and only the rewiring connections are visible. As $\mathscr{A}$ increases, the population 1 nodes (blue points) begin to form clusters with many connections between clusters. The black boxes are shown zoomed in on Fig. S2C. *Bottom Row:* The dynamics of V (WS-FHN) averaged across population 1 (blue) and 2 (black) are shown for a fixed value of $G = 0.253$, for strong coupling $\overline{g}_{coup} = 0.1$, and on each of the five iterations of the sorting algorithm. A phase transition from quiescence to global, synchronised activity occurs with respect to $\mathscr{A}$. **B.** *Top Row:* Five iterations of the sorting algorithm on the β-cell graph. Initially, the population 1 cells (blue) are spread uniform randomly throughout the lattice. As $\mathscr{A}$ increases, they begin to form localised clusters culminating in a single cluster at convergence ($a = 239$). *Bottom Row:* The dynamics of V (βC-FHN) averaged across population 1 (blue) and 2 (black) are shown for a fixed value of $G = 0.253$, for strong coupling $\overline{g}_{coup} = 0.1$, and on each of the five iterations of the sorting algorithm. A phase transition from quiescence to global, synchronised activity again occurs with respect to $\mathscr{A}$. **C.** The average number of peaks $\overline{P}$ as a function of $(G, \mathscr{A})$ for one run of the sorting algorithm on a WS-FHN network (*left*) and one run on the βC-FHN network (*right*) and for 20 equispaced values of $G \in [0.15, 0.345]$. As sortedness increases, the drive necessary for global, synchronised activation of the network decreases. figure.caption.7A. The initial network ($a = 0$) shows the population 1 nodes mostly isolated, but the final network ($a = 314$) shows two distinct clusters of several population 1 nodes.

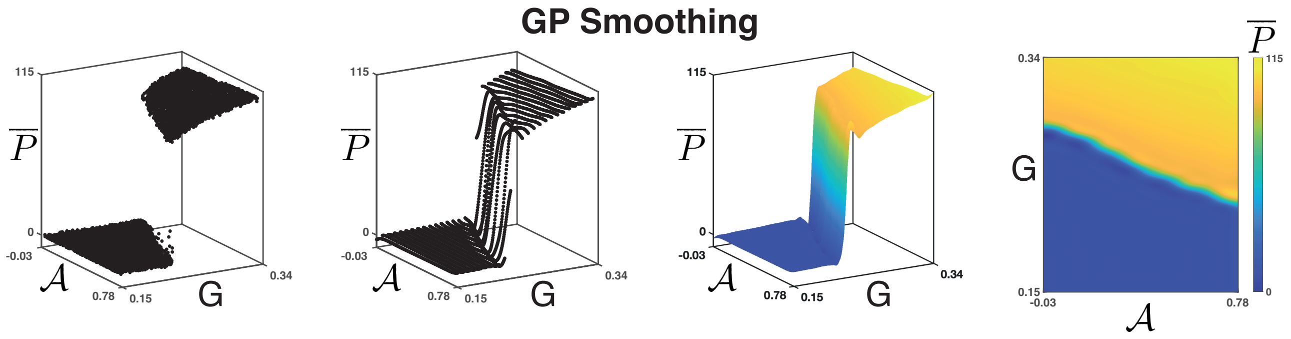

Figure S3. Gaussian process regression model. We now simulate dynamics on networks where (G, a) pairs are selected on a Latin hypercube. For each point, we use a different initial network P_k^0 and run the sorting algorithm to find the network P_k^a. We also use randomly selected initial conditions, $Y(0)$. In the case of the WS networks, we also use a different WS graph for each point. *Left panel* The feature $\overline{P}$ is shown as a scatter plot for each point $(G, \mathscr{A})$. *Middle-left panel* We use a Gaussian process regression model trained to the data on the Latin hypercube to estimate the points on a regular grid. *Middle-right panel* We can use this smoothed dataset to produce surface plots. *Right panel* We show the panels as heatmaps. Here, we show the resulting heatmap after training GP model with 10000 points using the WS-FHN network model. This is for the case where coupling is strong ($\overline{g}_{coup} = 0.1$).

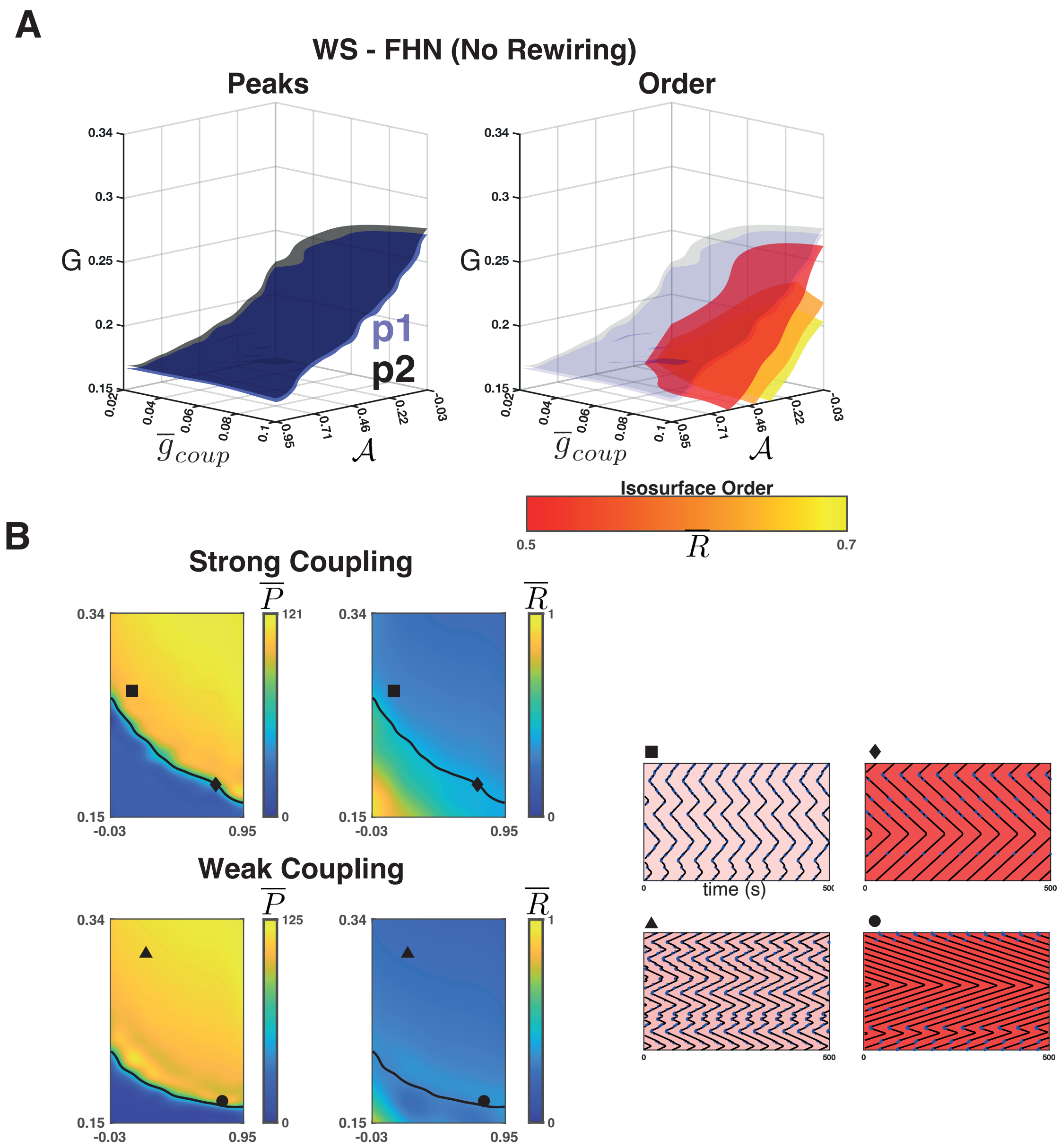

Figure S4. No rewiring in the WS-FHN networks. A. *Left panel:* The isosurfaces for the $\overline{P}_1$ (blue) and $\overline{P}_2$ (black) half-maxima in the WS-FHN networks with no rewiring. These surfaces do not separate. *Right panel:* The isosurfaces for $\overline{R}$ illustrate the boundary between low and high synchrony as well as its direction in the WS-FHN, $\beta = 0$ networks. Within the active region, synchrony is always low due to the propagation of waves. **B.** Heatmaps for $\overline{P}$ *(top left panel)* and $\overline{R}$ *(top right panel)* when $\overline{g}_{coup} = 0.1$ (strong). Heatmaps for $\overline{P}$ *(bottom left panel)* and $\overline{R}$ *(bottom right panel)* when $\overline{g}_{coup} = 0.02$ (weak). This shows that the phase transition is still a decreasing function of $\mathscr{A}$, but that synchrony is low regardless of coupling strength. *Square*, *diamond*, *triangle*, and *circle* show wave propagation for strong and weak coupling and low and high sortedness.

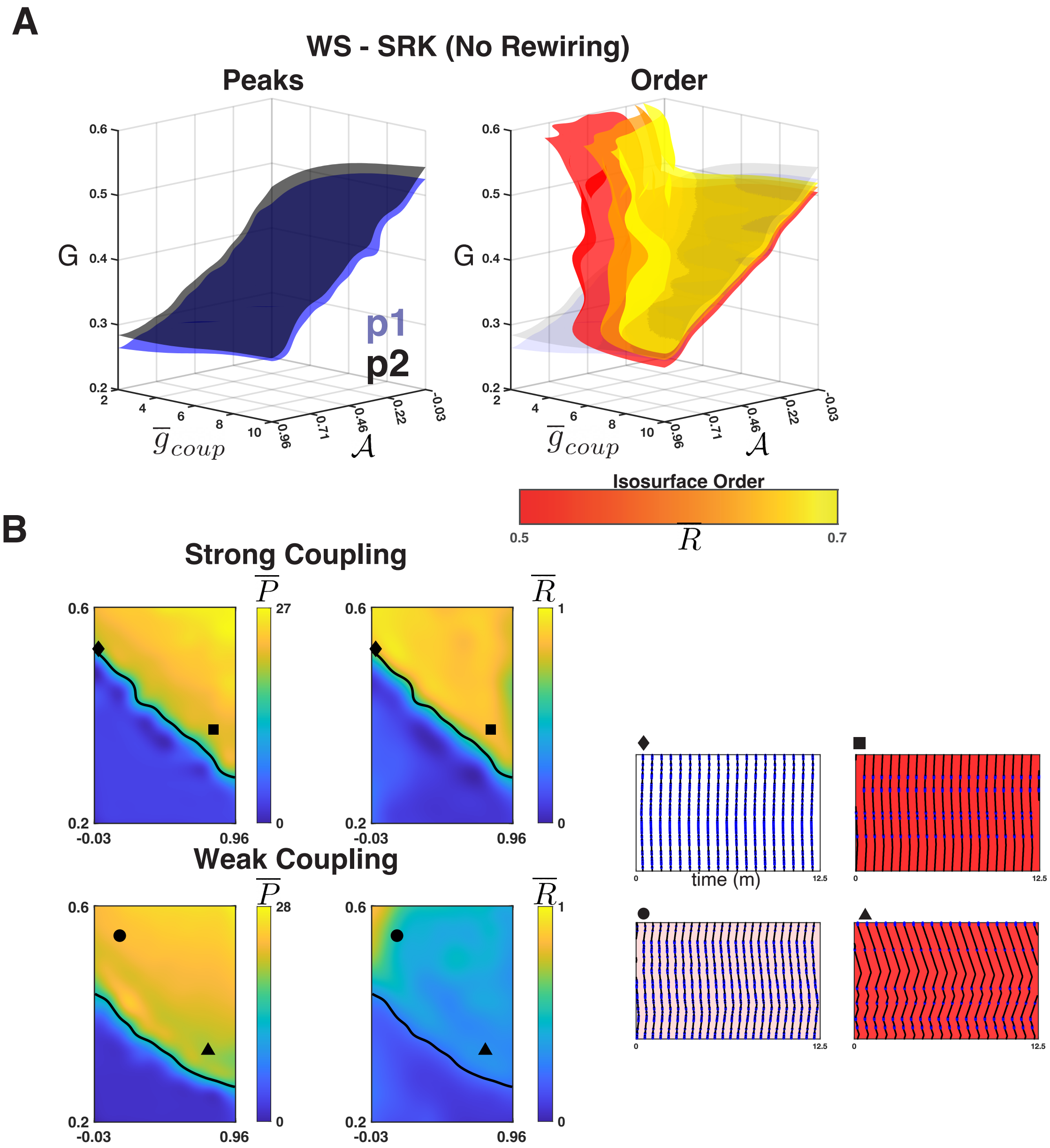

Figure S5. **No rewiring in the WS-SRK networks. A.** *Left panel:* The isosurfaces for the $\overline{P}_1$ (blue) and $\overline{P}_2$ (black) half-maxima in the WS-SRK networks with no rewiring. These surfaces do not separate. *Right panel:* The isosurfaces for $\overline{R}$ illustrate the boundary between low and high synchrony as well as its direction in the WS-SRK, $\beta = 0$ networks. Within the active region, synchrony is low for low to intermediate coupling due to wave propagation. **B.** Heatmaps for $\overline{P}$ *(top left panel)* and $\overline{R}$ *(top right panel)* when $\overline{g}_{coup} = 10$ (strong). Heatmaps for $\overline{P}$ *(bottom left panel)* and $\overline{R}$ *(bottom right panel)* when $\overline{g}_{coup} = 2$ (weak). This shows that the phase transition is still a decreasing function of $\mathscr{A}$, that synchrony is low for weak coupling, but that synchrony is still high for strong coupling. *Square* and *diamond* show synchronus activity for strong coupling. *Triangle*, and *circle* show wave propagation for weak coupling, but that the wave propagation remains fast relative to the period of *c*.

S1.2 Description of the routines used to generate a βC lattice network and perform the sorting algorithm on it

Algorithm 2-Algorithm 7 are used by Algorithm 1 which is described in the main text.

Algorithm 2 returns a set of points in $\mathbb{R}^3$ corresponding to the centres of spheres within a hexagonal close packed lattice (hcp). The input r_{ball} corresponds to the radius of the spheres within the lattice, which we set to $r_{ball} = 0.5$ so that the distance between any two nearest neighbors is $d_{ball} = 2r_{ball} = 1$. Algorithm 2 produces the hcp lattice using a sequence of scalings and shifts of a square lattice which takes the points $\{(x,y,z) \mid x,y,z \in \{1,\ldots,M\}\}$, where M is an integer corresponding to the number of spheres along the length of the lattice. We sought to embed a larger sphere, S_{net}, of radius R_{net} within the resulting hcp-lattice, and therefore, must choose M such that S_{net} is contained within the lattice. For the square lattice, a natural choice would be $M = 2R_{net}$, so that the length of the lattice equals the diameter of the sphere. However, for the hcp-lattice, the size of the resulting structure is $(M-1)x_{scale} + d_{ball} = Mx_{scale} = Md_{ball}$ by $(M-1)y_{scale} + d_{ball} > My_{scale}$ by $(M-1)z_{scale} + d_{ball} > Mz_{scale}$ (ignoring the shifts). To counteract this, we use:

$$M = \text{ceil}\left(\frac{2R_{net}}{\min([x_{scale}, y_{scale}, z_{scale}])}\right). \tag{S1}$$

We found that this choice of M generated a lattice which could fully embed the sphere, at least for our selection of $R_{net} = 5.55$ (in particular, we increased M and found that the number of nodes within the sphere did not increase).

Algorithm 3 first runs Algorithm 2 to produce an hcp-lattice. It then centres the lattice at the origin (i.e., at $(0,0,0)$) and finds all points that are within a sphere of radius R_{net} centred at the origin, which define the nodes in the network. It also returns N, the number of nodes in the spherical hcp-lattice ($N = 1,018$ in this work). Algorithm 4 establishes the Boolean adjacency matrix representing the connections between nodes in the spherical hcp-lattice. A connection exists between two nodes if they are at a distance of d_{ball} from one another. In other words, if two spheres (of radius r_{ball}) centred at the locations assigned to two nodes would be touching, then a connection exists between them. Algorithm 5 determines the population sets P_k for $k \in 1,2$. It returns the number of nodes N_k in each population, the population membership sets, and the initial network sortedness value A_0. Algorithm 6 determines the selection probabilities for every pair of nodes ($\{(i,j) \mid i \in P_1,\, j \in P_2\}$). Algorithm 7 chooses a candidate swap, produces the population sets established by that swap, and calculates $\mathscr{A}$ for the updated population sets.

Algorithm 1 Algorithm for producing networks

Inputs:

N: number of nodes in network

a: number of iterations of swapping algorithm to attempt

Dir: signed integer determining whether algorithm runs forwards (positive) or backwards (negative)

ρ: proportion of population 1 nodes

Outputs:

$\mathscr{A}$: network sortedness value

P_1: population 1 set

P_2: population 2 set

n: number of swaps performed

function GENERATENETWORK(N, a, Dir, ρ)
    (x, y, z), r, $K \leftarrow$ ESTABLISHLATTICE(N)
    N_1, N_2, P_1, P_2, $\mathscr{A} \leftarrow$ ASSIGNINITIALPOPULATIONS(N, ρ)
    $Term \leftarrow$ **false** ▷ Boolean determining whether terminal network state has been reached
    $n \leftarrow 0$
    while ($n < a$) and ($Term =$ **false**) **do**
        f, F, $Q \leftarrow$ COMPUTESELECTIONPROBABILITIES($r[]$, N_1, N_2, P_1, P_2)
        $m \leftarrow 0$
        $swap \leftarrow$ **true** ▷ Boolean determining whether to attempt swaps
        while ($m < N_1 \times N_2$) and ($swap =$ **true**) **do**
            $\widetilde{P}_1$, $\widetilde{P}_2$, $\mathscr{A}_p$, $k \leftarrow$NODESWAP(f, F, Q, Dir, N_1, N_2, P_1, P_2)
            if $\text{sgn}(\mathscr{A}_p - \mathscr{A}) = \text{sgn}(Dir)$ **then**
                P_1, $P_2 \leftarrow \widetilde{P}_1$, $\widetilde{P}_2$
                $\mathscr{A} \leftarrow \mathscr{A}_p$
                $n \leftarrow n + 1$
                $swap \leftarrow$ **false**
            else ▷ Reject swap if $\mathscr{A}$ does not change in the desired direction
                for $l \leftarrow k$ to $N_1 \times N_2$ **do**
                    $F[l] \leftarrow F[l] - f[k]$
                end for
                $Q \leftarrow Q - f[k]$
                $m \leftarrow m + 1$
            end if
        end while
        if $swap =$ **true then**
            $Term \leftarrow$ **true** ▷ Terminal state has been reached
        end if
    end while
    return $\mathscr{A}$, P_1, P_2, n
end function

Algorithm 2 Initialising HCP lattice

Inputs:

R_{net}: radius of the spherical lattice

r_{ball}: radius of balls around points in the lattice

Outputs:

$(x_1, y_1, z_1), \ldots, (x_N, y_N, z_N)$: (x, y, z) coordinates of nodes

N: number of nodes in hcp-lattice

function ESTABLISHHCPLATTICE(R_{net}, r_{ball})

$d_{ball} \leftarrow 2r_{ball}$

$x_{scale} \leftarrow d_{ball}$

$y_{scale} \leftarrow \sqrt{d_{ball}^2 - r_{ball}^2}$

$z_{scale} \leftarrow \sqrt{\frac{2}{3}} d_{ball}$

$x_{shift} \leftarrow r_{ball}$

$y_{shift} \leftarrow -\frac{d_{ball}}{\sqrt{3}}$

$M \leftarrow \text{ceil}(\frac{2R_{net}}{\min([x_{scale}, y_{scale}, z_{scale}])})$

$counter \leftarrow 0$

for $i \leftarrow 1$ to M **do**

for $j \leftarrow 1$ to M **do**

for $k \leftarrow 1$ to M **do**

$counter \leftarrow counter + 1$

$x[counter] \leftarrow k \times x_{scale}$

$y[counter] \leftarrow j \times y_{scale}$

$z[counter] \leftarrow i \times z_{scale}$

if j even **then**

$x[counter] \leftarrow x[counter] + x_{shift}$

end if

if i even **then**

$y[counter] \leftarrow y[counter] + y_{shift}$

end if

end for

end for

end for

$N \leftarrow M^3$ ▷ Total number of nodes in the lattice

return (x, y, z), N

end function

Algorithm 3 Initialising Sphere lattice

Inputs:

R_{net}: radius of the spherical lattice
r_{ball}: radius of points in the lattice

Outputs:

$(x_1, y_1, z_1), \ldots, (x_{N_{net}}, y_{N_{net}}, z_{N_{net}})$: $(x_{sphere}, y_{sphere}, z_{sphere})$ coordinates of nodes
$r_1, \ldots, r_{N_{net}}$: r_{sphere} radii of nodes
N_{net} number of nodes in the spherical lattice

function ESTABLISHSPHERELATTICE(R_{net}, r_{ball})
  $(x, y, z), N \leftarrow$ESTABLISHHCPLATTICE(R_{net}, r_{ball})
  $x \leftarrow x - \text{mean}(x)$ ▷ demean vector x
  $y \leftarrow y - \text{mean}(y)$ ▷ demean vector y
  $z \leftarrow z - \text{mean}(z)$ ▷ demean vector z
  $r \leftarrow \sqrt{x^2 + y^2 + z^2}$ ▷ compute norm over all points
  $counter \leftarrow 0$
  for $i \leftarrow 1$ to N **do**
    if $r[i] <= R_{net}$ **then** ▷ Find members of hcp-lattice within sphere radius R_{net}
      $counter \leftarrow counter + 1$
      $x_{sphere}[counter] \leftarrow x[i]$
      $y_{sphere}[counter] \leftarrow y[i]$
      $z_{sphere}[counter] \leftarrow z[i]$
      $r_{sphere}[counter] \leftarrow r[i]$
    end if
  end for
  $N_{net} \leftarrow counter$ ▷ Define number of nodes within the spherical domain
  return $(x_{sphere}, y_{sphere}, z_{sphere})$, r_{sphere}, N_{net}
end function

Algorithm 4 Initialising lattice

Inputs:

R_{net}: radius of the spherical lattice
r_{ball}: radius of points in the lattice

Outputs:

$(x_1, y_1, z_1), \ldots, (x_N, y_N, z_N)$: (x, y, z) coordinates of nodes
$r_1, \ldots, r_N$: r radial coordinate of nodes
$K \in \mathbb{R}^N \times \mathbb{R}^N$: connectivity matrix

function ESTABLISHLATTICE(R_{net}, r_{ball})
  $(x, y, z), r, N \leftarrow$ESTABLISHSPHERELATTICE($R_{net}, r_{ball}$)
  $d_{ball} \leftarrow 2r_{ball}$
  for $i \leftarrow 1$ to N **do**
    for $j \leftarrow 1$ to N **do**
      $dist \leftarrow \sqrt{(x[i] - x[j])^2 + (y[i] - y[j])^2 + (z[i] - z[j])^2}$
      if $dist = d_{ball}$ **then**
        $K[i][j] \leftarrow 1$
      else
        $K[i][j] \leftarrow 0$
      end if
    end for
  end for
  return (x, y, z), r, K
end function

Algorithm 5 Initialising populations

Inputs:

N: number of nodes in network

ρ: proportion of population 1 nodes

Outputs:

N_1, N_2: number of nodes in the respective population 1

P_1, P_2: population sets

$\mathscr{A}$: network sortedness

function ASSIGNINITIALPOPULATIONS(N, ρ)
  $U \leftarrow$ random permutation of $\{1, \ldots, N\}$
  $N_1 \leftarrow$ floor(ρN)
  $N_2 \leftarrow N - N_1$
  $P_1, P_2 \leftarrow$ integer array of length N_1, integer array of length N_2
  for $k \leftarrow 1$ to N_1 **do**
    $P_1[k] = U[k]$ ▷ Assign first N_1 elements of U to P_1
  end for
  for $k \leftarrow 1$ to N_2 **do**
    $P_2[k] = U[N_1 + k]$ ▷ Assign last N_2 elements of U to P_2
  end for
  $\mathscr{A} \leftarrow$ network sortedness value (17Definitionequation.2.17) using P_1 and P_2
  return N_1, N_2, P_1, P_2, $\mathscr{A}$
end function

Algorithm 6 Defining node pair selection probabilities

Inputs:

$r_1, \ldots, r_N$: radial coordinates of nodes

N_1, N_2: number of nodes in the respective population

P_1, P_2: population sets

Outputs:

$f \propto$ probability density function for node pair selection

$F \propto$ cumulative density function for node pair selection

Q: normalisation constant for f

function COMPUTESELECTIONPROBABILITIES($r[]$, N_1, N_2, P_1, P_2)
  $f \leftarrow$ array of length $N_1 \times N_2$,
  $F \leftarrow$ array of length $N_1 \times N_2 + 1$
  $F[1] \leftarrow 0$
  $k, Q \leftarrow 0$
  for $i \leftarrow 1$ to N_1 **do**
    for $j \leftarrow 1$ to N_2 **do**
      $k \leftarrow k + 1$
      $p \leftarrow 1/R_{n_i,P_1} \times 1/R_{n_j,P_2}$ ▷ Weight probability of node pair being selected using (20Node selection probabilitiesequation.2.20)
      $f[k] = p$
      $Q \leftarrow Q + p$
      $F[k] \leftarrow Q$
    end for
  end for
  return f, F, Q
end function

Algorithm 7 Node population swapping

Inputs:

$f \propto$ probability density function for node pair selection

$F \propto$ cumulative density function for node pair selection

Q: normalisation constant for f

P_1, P_2: sets of indices of nodes in the respective population

Outputs:

$\widetilde{P}_1, \widetilde{P}_2$: population sets following node population swap

$\mathscr{A}_p$: network sortedness of network with node populations swapped

k: index of node pair swapped

function NODESWAP($f, F, Q, N_1, N_2, P_1, P_2$)
  $u \leftarrow U(0,1)$ ▷ Sample from unit uniform distribution
  $k \leftarrow 1$
  while $u < F(k)/Q$ **do**
    $k \leftarrow k+1$
  end while
  $i, j \leftarrow k/N_2, (k-1) \bmod N_2 + 1$ ▷ Indices of selected population nodes
  $\widetilde{P}_1, \widetilde{P}_2 \leftarrow P_1, P_2$ ▷ Create copies of P_1 and P_2
  $\widetilde{P}_1(i), \widetilde{P}_2(j) \leftarrow P_2(j), P_1(i)$ ▷ Trial node population swap
  $\mathscr{A}_p \leftarrow$ network sortedness value (17Definitionequation.2.17) using $\widetilde{P}_1$ and $\widetilde{P}_2$
  return $\widetilde{P}_1, \widetilde{P}_2, \mathscr{A}_p, k$
end function

S1.3 Evaluation of collective dynamics

For each node, the number of peaks was identified by searching for maxima with a peak prominence of 0.02 μM in the Ca^{2+} timecourse (SRK) or 2 a.u. in the v timecourse (FHN) across the simulation duration.

For a network with N nodes, the time-dependent Kuramoto order parameter is a complex-valued scalar defined as

$$z(t) = R(t)\mathrm{e}^{i\Theta(t)} = \frac{1}{N}\sum_{j=1}^{N} \mathrm{e}^{i\theta_j(t)}, \tag{S2}$$

where $\theta_j(t)$ is the phase of the jth node, as extracted via a mean-subtracted Hilbert transform of the Ca^{2+} signal (SRK) or v signal (FHN) for node j. The argument of z, Θ, is the mean phase of the network whilst its magnitude, R, measures the degree of synchrony across the network. We sample the Ca^{2+} at equispaced time points $t_i = i\delta t$, $i = 0, \dots T-1$ and record the time-averaged degree of synchronisation: $\overline{R} = \frac{1}{T}\sum_{i=0}^{T-1} R(t_i)$.

REFERENCES

[1] Bard Ermentrout. *Simulating, Analysing and Animating Dynamical Systems: A Guide to XPPAUT for Researchers and Students*. SIAM, 2002.
